# Research progress on antitumor mechanisms and molecular targets of *Inula* sesquiterpene lactones

**DOI:** 10.1186/s13020-023-00870-1

**Published:** 2023-12-18

**Authors:** Fei Cao, Chu Chu, Jiang-Jiang Qin, Xiaoqing Guan

**Affiliations:** 1 College of Pharmaceutical Sciences, Zhejiang University of Technology, Hangzhou, Zhejiang China; 2 Zhejiang Cancer Hospital, Hangzhou Institute of Medicine (HIM), Chinese Academy of Sciences, Hangzhou, 310022 Zhejiang China; 3 Key Laboratory of Prevention, Diagnosis and Therapy of Upper Gastrointestinal Cancer of Zhejiang Province, Hangzhou, Zhejiang China

**Keywords:** *Inula* sesquiterpene lactones, Antitumor, Structure–activity relationship, Mechanisms of action, Molecular target

## Abstract

The pharmacological effects of natural product therapy have received sigificant attention, among which terpenoids such as sesquiterpene lactones stand out due to their biological activity and pharmacological potential as anti-tumor drugs. *Inula* sesquiterpene lactones are a kind of sesquiterpene lactones extracted from *Inula* species. They have many pharmacological activities such as anti-inflammation, anti-asthma, anti-tumor, neuroprotective and anti-allergic. In recent years, more and more studies have proved that they are important candidate drugs for the treatment of a variety of cancers because of its good anti-tumor activity. In this paper, the structure, structure–activity relationship, antitumor activities, mechanisms and targets of *Inula* sesquiterpene lactones reported in recent years were reviewed in order to provide clues for the development of novel anticancer drugs.

**Graphical abstract:**

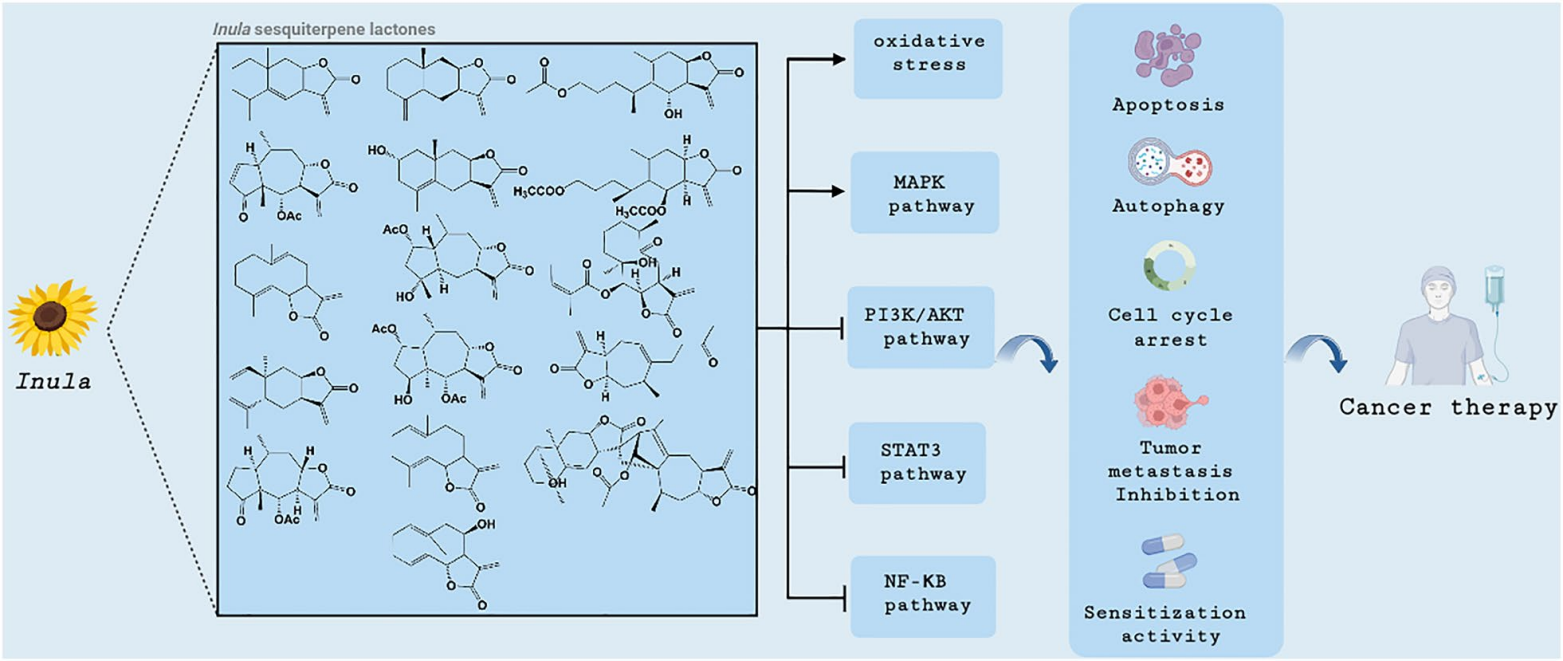

## Introduction

An important source of antitumor drugs is natural products and their derivatives, which are rich in sources and novel in structure, providing many active molecules for the research and development of antitumor drugs [1]. At present, a number of molecularly targeted antitumor drugs derived from natural products or their derivatives have been used in tumor therapy, such as paclitaxel, vincristine, and camptocampin. Therefore, the development of anti-tumor drugs based on natural products has good prospects.

As one of the natural products, the antitumor activity and mechanism of *Inula* have also attracted extensive attention. *Inula* belongs to the family Composite. There are approximately 100 species of this genus in the world, some of which are widespread species and some are endemic, mainly distributed in Europe, Africa and Asia [2]. It was first reported in “Shennong's Herbal Classic” and has a long medicinal history. In the 2020 edition of the "Pharmacopoeia of the People's Republic of China", the capitula of *Inula japonica Thunb*. and *l.britannica* L. were included as authentic *Inula*. *Inula* contains rich chemical components, mainly including sesquiterpenes, flavonoids, volatile oils, polysaccharides, steroids, etc. [3]. Among them, sesquiterpenoids, especially sesquiterpenoid lactones, are the characteristic components of *Inula* and have significant biological activities [4].

Sesquiterpene lactones are of great interest because they show great structural diversity and a wide range of biological activities, including anti-inflammatory, anti-tumor, anti-microbial, and antiviral effects [5]. Artemisinin [6], parthenolide [7] and thapsigargin [8] are representative sesquiterpene lactones, which are promising anticancer drugs. Similarly, with the development of research, the anti-tumor activity of *Inula* sesquiterpene lactones has received more and more attention from researchers. They have been found to have a good therapeutic effect on a variety of cancers. However, there is little literature summarizing the latest research progress on their anti-tumor effects. Therefore, this review provides a comprehensive overview of the antitumor activity of *Inula* sesquiterpene lactones, as well as its mechanism and target of action, to provide a reference for further investigation by researchers.

## Structure of *Inula* sesquiterpene lactones

### Structure of *Inula* sesquiterpene lactones

**Fig. 1:**
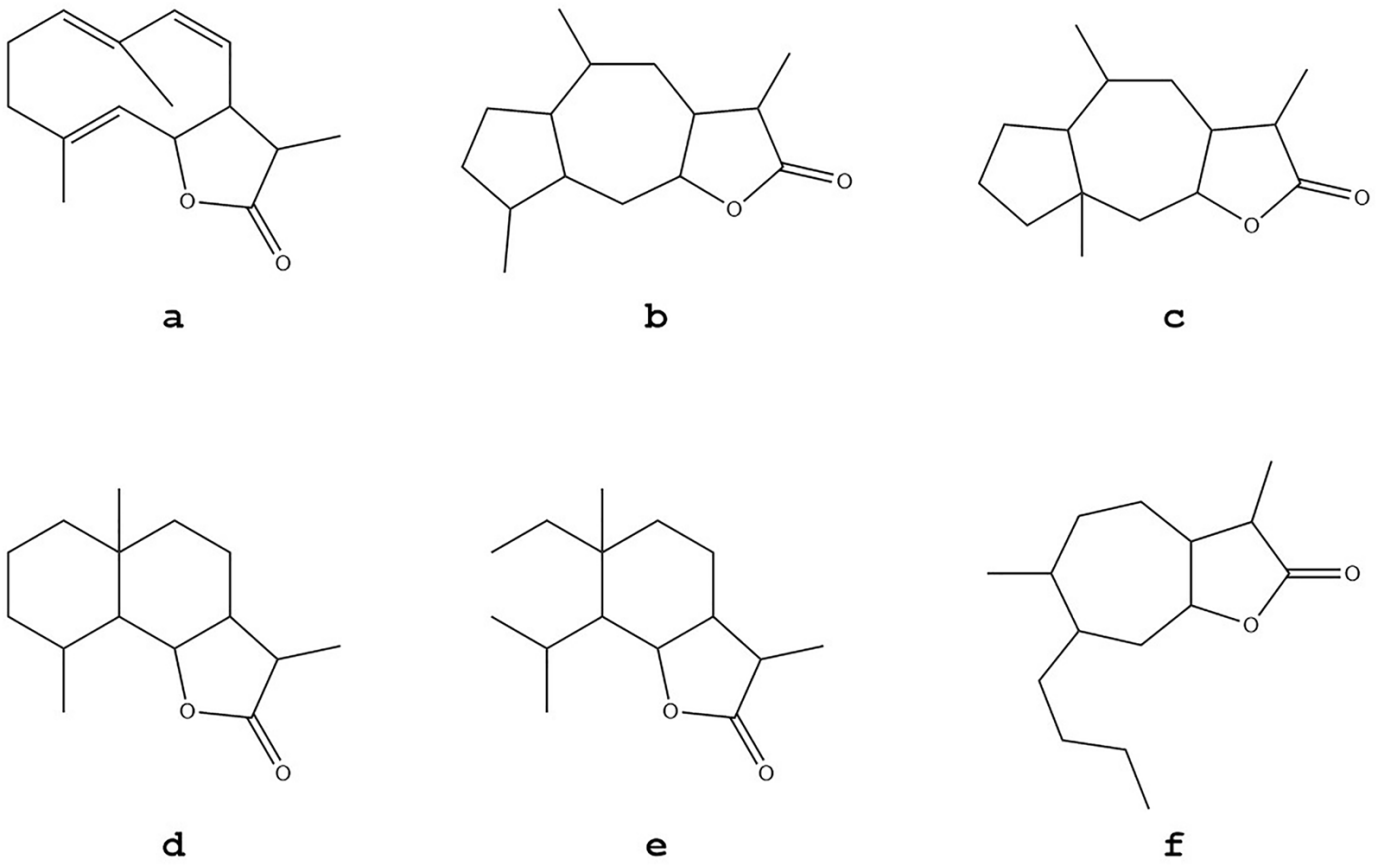
Classification of sesquiterpene lactones (**a** germacranolides, **b** guaianolides, **c** pseudoguaianolides, **d** eudesmanolides, **e** xanthanolide, **f** elemanolides)

**Fig. 2:**
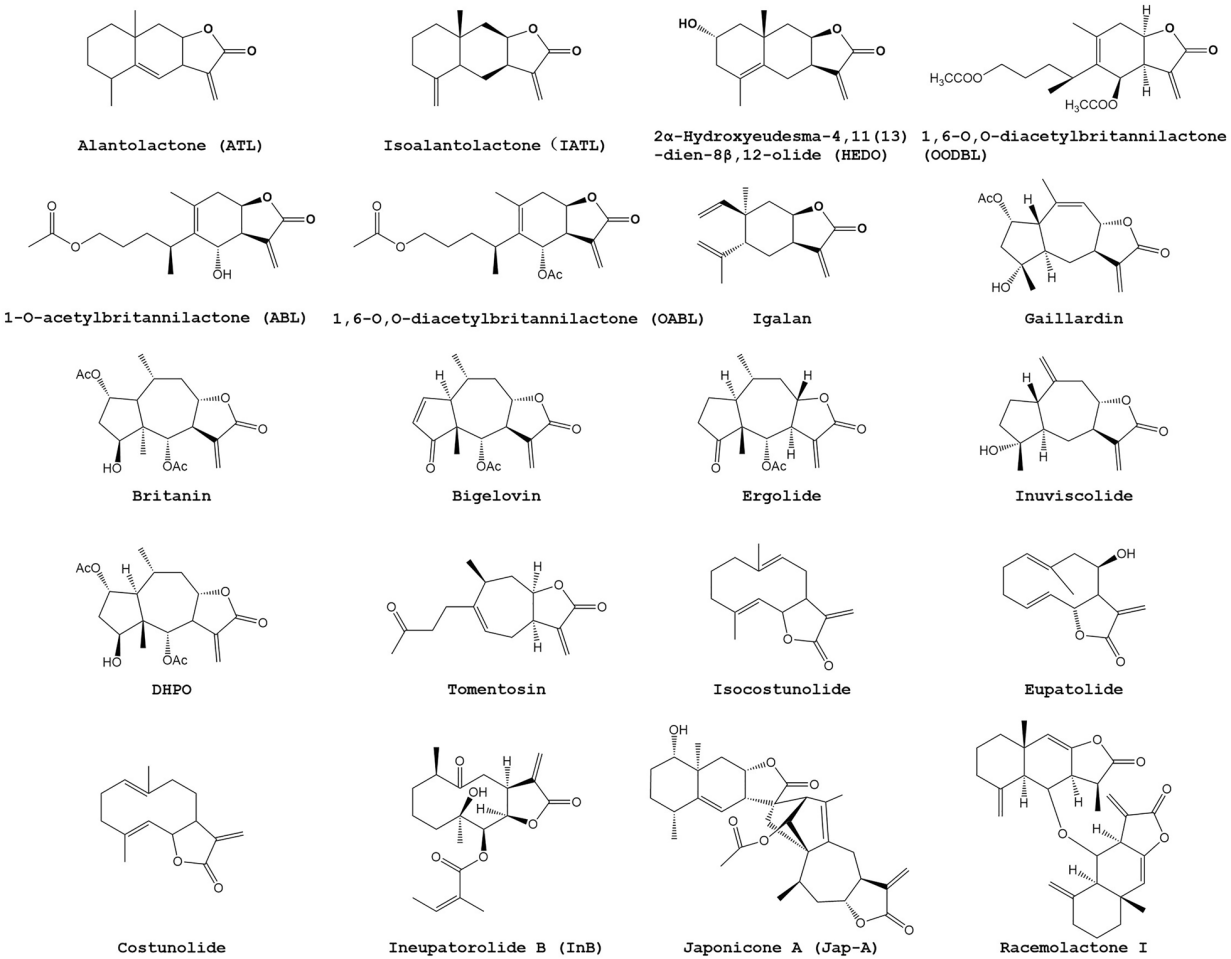
Structures of *Inula* sesquiterpene lactones possessing antitumor activity

The chemical structure of sesquiterpene lactones is based on a skeleton of fifteen carbon atoms and consists of three cyclic isoprene structures, one of which is a pentamembered (γ) lactone group (cyclic ester) [9]. Based on the type and location of its carboxyl skeleton and substituents, sesquiterpene lactones can be divided into germacranolides, guaianolides, pseudoguaianolides, eudesmanolides, xanthanolides and elemanolides (Fig. 1). In Fig. 2, we list the structures of several important and representative *Inula* sesquiterpene lactones with antitumor activity.

### Structure–activity relationship of *Inula* sesquiterpene lactones

#### α-methylene-gamma-butyrolactone structure is essential for cytotoxic activity

**Fig. 3:**
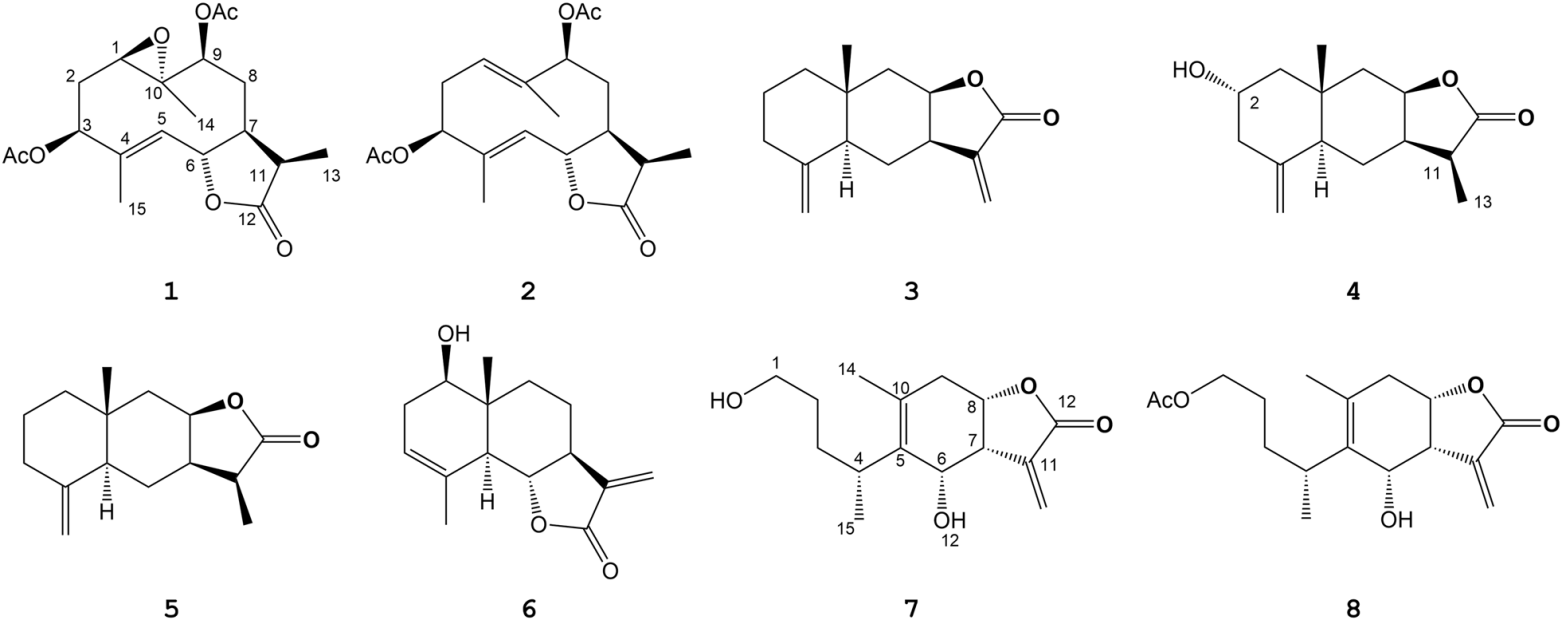
Chemical structures of compounds 1–8 isolated from* Inula*

Chitosan derivatives containing an α-methylene-γ-butyrolactone skeleton have obvious biological activity, especially antitumor activity. Due to the double bond action of α-methylene-γ-butyrolactone, it can react as an electrophilic group with nucleophilic groups on the basic groups of some active sites in the organism, thereby changing the structure of these active sites and showing different biological effects. It plays an important role in cell growth through the reaction of α-methylene-γ-butyrolactone with sulfhydryl (-SH), which may be a potential cytotoxic mechanism of sesquiterpene lactones containing α-methylene-γ-butyrolactone rings [10]. There was a study that supports this conclusion. Eight sesquiterpene lactones were isolated from the genus *Inula*, among which compounds 1 and 2 were germacranolides with a 10-membered ring, compounds 3–6 were eudesmanolides with a transdecalin (6/6-membered) ring and compounds 7 and 8 were xanthanolides with a 6-membered ring (Fig. 3). The results showed that their cytotoxic activity of human lung cancer cells was closely related to the carbon skeleton and γ-ectomylene in the α-lactone ring. The saturated of α-ecomethylene or cleavage of 6/6-membered ring may result in loss or reduction of cytotoxic activity [11]. Similarly, another result also reached the same conclusion that if C-11 and C-13 were saturated, the antitumor activity of the compound would be significantly reduced [12].

#### 6-OH modification can increase cytotoxic activity

1-O-acetylbritannilactone (ABL) and 1,6-O,O-diacetylbritannilactone (OABL), two sesquiterpene lactones extracted from *Inula britannica* L. Han et al. modified the 6-OH position of ABL and synthesized 19 analogues. The relationship between pharmacological activity and structure–activity of ABL was studied. It was found that when the 6-OH site was acetylated or appropriate lipophilic aliphatic chain was introduced, the cytotoxic effect was significantly enhanced. Moreover, when the length of the introduced aliphatic side chain was 12C, the cytotoxicity was the highest. This suggests that the introduction of appropriately enhanced lipophilic aliphatic chains at 6-OH of ABL leads to increased activity and the 12C aliphatic side chain may be the optimal length for cytotoxic activity [13].

#### Arylation of C-13 can reduce the cytotoxic activity

Five OABL arylation analogues were synthesized by Heck coupling reaction of OABL with readily available aryl iodide, that is, aryl was introduced into the α-methylene-γ-lactone motif of OABL to reduce the nucleophilic activity of α-methylene-lactone. The results showed that the cytotoxicity of these 5 arylated analogues was decreased [13].

## Antitumor activity of *Inula* sesquiterpene lactones

Various molecular, cellular and animal studies have shown that *Inula* sesquiterpene lactones can inhibit the growth and metastasis of human cancer cells, induce apoptosis, autophagy and cell cycle arrest, and increase the sensitivity of chemotherapy drugs (summarized in Table 1). Their apparent antitumor activity could encourage us to use them as potential treatments for oncologic diseases, as well as to search for more and more effective natural products of *Inula* sesquiterpene lactones.

**Table 1:** The antitumor mechanisms of action of different *Inula* sesquiterpene lactones

Compounds	Cancers	In vitro Activities	In vivo activity	Mechanisms	Targets	Refs.
Gaillardin	Gastric cancer	Inhibit cell viability (IC50 = 42–52 µM) and induce apoptosis	NR	Inhibit the activation of NF-κB and down-regulate genes regulated by NF-κB	NR	[56]
	Breast cancer	Inhibit cell viability (IC50 = 4.9 and 5.5 µM) and induce apoptosis	NR	Induce mitochondrial apoptotic pathway, upregulate the pro-apoptotic protein Bax and p53 and down-regulate Bcl-2	NR	[17]
	Leukaemia	Inhibit cell viability (IC50 = 4.3 and 6.1 µM) and induce apoptosis, extend chemotherapeutic sensitivity	NR	Increase in the expression of pro-apoptotic genes such as Bax and Caspase-3, decrease the expression of BCl-2	NR	[117]
Bigelovin	Colorectal cancer	Inhibit cell viability (IC50 = 0.8 and 1.2 µM), induce apoptosis, inhibit inflammation and angiogenesis	Inhibited liver/lung metastasis (liver metastasis from 19.0% to 9.5% and lung metastasis from 19.2% to 13.1% at 1 mg/kg) in orthotopic colon tumor allograft-bearing mice	Interfere IL6/STAT3 and cofilin pathways	NR	[35]
	Pan-cancer	Inhibit cell viability (IC50 = 0.8–10.0 µM) and induce apoptosis	NR	Inhibite the JAK2/STAT3 pathway through directly interacting with JAK2	JAK2	[65]
	Colon cancer	Inhibit cell viability (IC50 = 5 µM) and induce apoptosis	NR	Induce IKK-β degradation and suppress NF-κB activation	NR	[55]
	Liver cancer	Induce apoptosis and autophagy	Inhibits tumor growth in HepG2 xenograft tumor mode at 5/10/20 mg/kg	Enhance accumulation of autophagosomes, increase LC3B-II and Beclin-1 and decrease p62	NR	[41]
	Colorectal cancer	Inhibit cell viability (IC50 = 37–41 µM) and induce cell cycle arrest and apoptosis	Reduce tumor growth (about 30% of tumor volume and 60% of tumor weight at 20 mg/kg) in HCT116 xenograft model	Up-regulate DR5 and increase ROS, cause DNA damage through regulating p21, p-Rb and p-H2AX expressions	NR	[118]
	Liver cancer	Inhibite the proliferation, migration and EMT	Reduce tumor growth (about 70% of tumor volume at 20 mg/kg) and lung metastasis in subcutaneous xenograft tumor and tail vein-injected lung metastases mouse models	Activate the MAPT-mediated Fas/FasL pathway	NR	[119]
Alantolactone(ATL)	Breast cancer	Inhibit cell viability (IC50 = 7.6–20.6 µM) and induce apoptosis	NR	Trigger the mitochondrial-mediated caspase cascade apoptotic pathway	NR	[18]
	Lung cancer	Induce apoptosis	NR	Activate p38 MAPK pathway and inhibit NF-κB pathway	NR	[53]
	Gastric cancer	Induce apoptosis and inhibit migration	NR	Activate p38 MAPK pathway and inhibit NF-κB pathway	NR	[54]
	Pancreatic cancer	Induce apoptosis and autophagy, improve chemosensitivity	Reduce tumor growth (about 66.7% of tumor volume at 3 mg/kg) in Panc1 xenograft model	Impair autophagy degradation leads to the accumulation of autophagosomes and reduce the level of TFEB	NR	[42]
	Gastric cancer	Induce apoptosis	Reduce tumor growth at 15 mg/kg in SGC7901 xenograft model	Inhibit TrxR1 activity, induce the production of ROS and activate the p38 MAPK pathway	NR	[79]
	Esophagus cancer	Induce apoptosis and inhibit invasion	NR	Regulate the Wnt/β-catenin signaling pathway	NR	[34]
	Lung cancer	Enhance the sensitivity of gemcitabine	NR	Increase ROS levels, inhibit AKT/GSK3β pathway and induce ER stress	NR	[47]
	Colorectal cancer	Inhibit cell viability (IC50 = 21.63 and 18.14 μM) and induce cell apoptosis and block cell cycle	NR	Increase ROS levels and the accumulation of cellular oxidized guanine (8-oxoG), resulting in oxidative DNA damage	NR	[81]
	Esophagus cancer	Inhibit cell viability (IC50 = 2.26-4.58 μM) and induce apoptosis	Reduce tumor growth (about 62.5% or 87.5% of tumor volume and 54.5% or 81.8% of tumor weight at 6 or 15 mg/kg) in KYAE-1 xenograft model	Increase NRF2- mediated ROS	NR	[87]
	Human monocytic leukemia	Inhibit cell viability and induce apoptosis	NR	Inhibit STAT3 and survivin expression	NR	[64]
	Liver cancer	Inhibit cell viability and proliferation (IC50 = 10 μM) and induce cell cycle arrest and apoptosis	NR	Suppress ROS-mediated AKT signalling and inhibit PINK1-mediated mitophagy	NR	[70]
2α-Hydroxyeudesma-4,11(13)-dien-8β,12-olide (HEDO)	Lymphoma	Induce apoptosis and block cell cycle	NR	Upregulate intracellular ROS and increase the depolarization of mitochondrial membrane potential	NR	[85]
Isoalantolactone (IATL)	Hepatocellular carcinoma	Inhibit cell proliferation	NR	Induce the activity of phase 2 enzyme by stimulating the accumulation of NRF2 in the nucleus	NR	[86]
	Breast cancer	Inhibit the migration and invasion	NR	Suppress the p38 MAPK/NF-κB signaling pathway	NR	[36]
	Osteosarcoma	Inhibit cell viability (IC50 = 40–50 µM) and induce apoptosis	NR	Upregulate DR-5, FADD and cleaved caspase-8, increase the interaction between DR-5 and FADD	NR	[52]
	Hepatocellular carcinoma	Induce apoptosis	NR	Activate the ROS-dependent JNK signaling pathway	NR	[67]
	Esophageal cancer	Inhibit cell viability (IC50 = 20–50 µM) and induce apoptosis	Reduce tumor growth (31.2% or 49.2% of tumor volume and 34.5% or 54.8% of tumor weight at 40 mg/kg or 80 mg/kg) in human esophageal cancer xenograft	Activate caspases-3, -7, and -10, and upregulate DR-5	NR	[22]
	Colon cancer	Enhance the cytotoxicity of doxorubicin	NR	Increase ROS production, induce DNA damage and activate the JNK signaling pathway	NR	[48]
Britanin	Pancreatic cancer	Inhibit cell viability (IC50 = 1.35–3.10 µM), proliferation and migration	Reduce tumor growth (about 50.0% or 71.4% of tumor volume and 44.4% or 66.7% of tumor weight at 5 or 10 mg/kg) in Panc-1 xenograft model	Suppress NF-κB activation	NR	[58]
	Liver cancer	Inhibit cell viability (IC50 = 6.86–27.86 µM) and induce apoptosis and autophagy	Reduce tumor growth (about 75.0% of tumor volume and 80.0% of tumor weight at 30 mg/kg) in HepG2 xenograft model	Support by the up-regulation of LC3 II, p62, ATG5 and Beclin-1	NR	[43]
	Hepatocellular carcinoma	Inhibit cell viability (IC50 = 2.702 and 6.006 µM) and suppress metastasis	Reduce tumor growth in BEL7402 subcutaneous tumor model	Inhibit p65 protein expression and reduce the ratio of Bcl-2/Bax	NR	[38]
	Breast cancer	Inhibit cell viability (IC50 = 29.37–52.65 µM) and suppress the invasion and metastasis	Reduce the macrometastases and micrometastases at 5 and 10 mg/kg in experimental lung metastasis model	Promote the degradation of ZEB1 protein, and thus down-regulate the protein expression levels of ZEB1, MMP-9 and CD44	NR	[37]
	Gastric cancer	Inhibit cell viability (IC50 = 2.243 and 4.999 µM) and suppress the proliferation	Reduce tumor growth at 5 and 10 mg/kg in BGC823 xenograft model	Affect the NF-κB signalling pathway	NR	[57]
	Colorectal cancer	Inhibit cell proliferation and endothelial cell angiogenesis	Reduce tumor growth at 5 and 15 mg/kg in HCT116 xenograft model	Inhibit the expression of PD-L1 by blocking the interaction between HIF-1α and MYC	PD-L1	[108]
	NR	Induce the expression of protective enzymes and reverse oxygen–glucose deprivation	Ameliorate middle cerebral artery occlusion-reperfusion (MCAO-R) insult	Bind to cysteine 151 of Keap1 and inhibit ubiquitination of NRF2, leading to induction of the NRF2 pathway	Keap1	[96]
Japonicone A(Jap-A)	Burkitt lymphoma	Inhibit cell viability (IC50 = 400–800 nM) and induce apoptosis and cell-cycle arrest	Reduce tumor growth (65.4% of tumor volume at 30 mg/kg) in both localized and disseminated lymphoma xenografts models	Inhibit TNF-α-stimulated NF-κB activity and nuclear translocation	NR	[59]
	NR	Reduce the TNF-α-mediated cytotoxicity	Reduce inflammation and liver damage at 8 mg/kg on TNF-α/D-GalN-induced hepatitis model	Directly bind to TNF-α and inhibit the binding of TNF-α to TNFR1, block TNF-α-triggered multiple signaling pathways	TNF-α	[88]
	Breast cancer	Inhibit cell viability (IC50 = 0.5–2.0 µM) and induced cell cycle arrest and apoptosis	Reduce tumor growth (77% and 87% of tumor volume at 15 and 30 mg/kg) in breast cancer xenograft models	Directly bind to MDM2 protein and reduced MDM2 levels by promoting MDM2 protein degradation and inhibiting MDM2 transcription	MDM2	[95]
Ergolide	Leukemia	Inhibit cell viability (IC50 = 3.56 and 4.21 µM) and induce apoptosis	NR	Inhibit the NF-κB signaling pathway	NR	[61]
	Cervical cancer	Induce apoptosis	NR	Inhibit NF-κB-dependent gene transcription	NR	[60]
	Pan-cancer	Induce cell cycle arrest and apoptosis, potentiate vincristine cytotoxicity in all cell lines	NR	Induce cell death through ROS-dependent manner by altering the expression of pro apoptotic related genes	NR	[46]
	NR	Induce pyroptosis	Improves the survival of septic mice and attenuate NLR family pyrin domain-containing 3 (NLRP3)-dependent acute lung injury in wild-type mice	Directly target the NLRP3 and NLRP3 NACHT domains, thereby providing anti-pyroptosis	NLRP3	[100]
1,6-O,O-diacetylbritannilactone (OODBL)	Leukaemia	Inhibit cell viability (IC50 = 18 µM) and induce apoptosis and cell cycle arrest	NR	Produce ROS, activate MAPK and JNK signaling pathways	NR	[68]
Igalan	Liver cancer	Activate cellular defense mechanisms and a detoxifying agent	NR	Activate the NRF2 pathway by increasing the inactive form of GSK3β, the phosphorylated form of AKT, and the nuclear accumulation of NRF2	NR	[71]
Costunolide	Leukaemia	Enhance sensitivity to doxorubicin	NR	Inhibit PI3K/AKT pathway, activate caspases 3 and downregulate the expression of p-glycoprotein	NR	[73]
	Breast cancer	Inhibit cell viability and proliferation	NR	Act as a microtubule-interacting agent and stimulate tubulin assembly	NR	[120]
Isocostunolide	Melanoma	Inhibit t cell viability (IC50 = 2–5 µg/ml) and induce apoptosis	NR	Induce a depolarization of mitochondrial membranes to facilitate cytochrome c release into cytosol	NR	[19]
Eupatolide	Breast cancer	Induce cycle arrest and apoptosis	NR	Down-regulate c-FLIP expression through the inhibition of AKT phosphorylation	NR	[74]
	Breast cancer	suppress the migration and invasion	NR	downregulat SMAD3 phosphorylation and transcriptional repression of TGF-β receptor 1 (ALK5), block the ERK and AKT signaling pathways	NR	[121]
	Lung cancer	Inhibit cell viability (IC50 = 10–18 µM) and enhance the activity of cisplatin and 5-FU	Inhibits tumor growth (about 75% or 50% of tumor volume and 75% or 50% of tumor weight at 10 mg/kg) in A549 or H1975 xenograft model	Inhibit the activation of STAT3	NR	[122]
Compound 24	Lung cancer	Inhibit paclitaxel-resistant cell viability (IC50 = 0.34 µM) and induce apoptosis	NR	Inhibit the expression of ABCC1, ABCG2 and MDR1 proteins	NR	[49]
Tomentosin	Leukemia	Inhibit cell viability (IC50 = 10 µM) and proliferation and induce apoptosis	NR	Inhibit mTOR/PI3K/AKT signaling pathway	NR	[75]
	Cervical cancer	Inhibit cell viability (IC50 = 7.1 and 5.9 µM) and induce cell cycle arrest and apoptosis	NR	Increase ROS and decrease Bcl-2 expression	NR	[25]
	Osteosarcoma	Inhibit cell viability (IC50 = 40 µM) and induce apoptosis	NR	Increase ROS, induce FOXO3 and p27 overexpression, decrease peroxiredoxin-1	NR	[123]
	Gastric cancer	Inhibit cell viability (IC50 = 20 µM) and induce cell cycle arrest and apoptosis	NR	increase ROS, downregulate BCl-2 and Cyclin D1 and inhibit inflammation	NR	[124]
Inuviscolide	melanoma	Inhibit cell viability (IC50 = 37–41 µM) and induce cell cycle arrest and apoptosis	NR	Activate ATM/R and decrease the expression of survivin and NF-kB	NR	[26]
1-O-acetylbritannilactone (ABL)	Laryngocarcinoma	Induce apoptosis	NR	Induce a tumor suppressor p53 and its target genes expression	NR	[114]
HFIH	Breast cancer	Induce apoptosis	Inhibit tumor growth (about 50% of tumor volume at 10 mg/kg) in human breast xenograft tumors	Suppress STAT3 phosphorylation at tyrosine 705	NR	[63]
Racemolactone I	Cervical cancer	Inhibit cell viability (IC50 = 0.9 µg/ml)	NR	Bind and interact with ATP-binding site of PLK1 and key amino acid residues, respectively	PLK1	[103]

NR, not reported

### Induction of apoptosis

Apoptosis is a form of programmed cell death. Many studies have found that apoptosis pathways include extrinsic pathways, intrinsic pathways (also known as mitochondria-mediated apoptosis pathways) and endoplasmic reticulum stress (ERS) pathways [14]. At present, intrinsic apoptosis is a relatively clear signaling pathway, mainly through the mitochondrial pathway. P53 is a transcription factor of outer mitochondria that can directly interact with proapoptotic protein Bax, change the ratio of Bax to Bcl-2, destroy the mitochondrial membrane, release proteins in mitochondria, and promote apoptosis of tumor cells [15]. Extrinsic receptor-mediated apoptosis is caused by the activation and connection of death receptors of the tumor necrosis factor family. The death receptors include Fas receptor, DR and TRAIL receptor. They activate the apoptotic proteases caspase-8 and caspase-10 near the membrane, which introduce death signals into cells and accelerate apoptosis [16].

Gaillardin is a guaiane type sesquiterpene lactone isolated from the plant *Inula oculus-christi*. Gaillardin has been shown to be a promising molecule in cancer chemoprophylaxis or chemotherapy, that can inhibit the proliferation of breast cancer cells by inducing the mitochondrial apoptotic pathway. Gaillardin can upregulate the proapoptotic proteins Bax and p53 and downregulate the anti-apoptotic protein Bcl-2 [17]. The same mechanism of action has been found in alantolactone (ATL), a natural sesquiterpene lactone originating from *Inula helenium L*. ATL can also trigger a mitochondria-mediated caspase cascade apoptotic pathway, which is demonstrated by increased Bax/Bcl-2 ratios and cell release from mitochondria to cytoplasm [18]. In addition, isocostunolide, a sesquiterpene lactone isolated from the roots of *Inula helenium.*, has also been reported to significantly induce depolarization of the mitochondrial membrane to promote the release of cytochrome c into the cytoplasm, thereby activating the mitochondria-mediated apoptosis pathway [19].

Isoalantolactone (IATL) is one of the major total sesquiterpene lactones isolated from *Inula helenium L*. [20], which has anti-inflammatory, antioxidant and neuroprotective pharmacological effects. Previous studies have shown that it may have potential in the prevention and treatment of neurodegenerative diseases, inflammatory diseases, and antitumor [21]. IATL has been found to activate caspase -3, caspase -7, and caspase -10 and upregulate the DR-5. If DR-5 is knocked down, the effect of IATL is partially reversed. These results suggest that IATL can induce exogenous cell apoptosis [22].

### Interference with the cell cycle

The cell cycle is a highly regulated process that promotes cell growth, replication of genetic material and cell division. The cell cycle regulatory system consists of a class of genes that are directly or indirectly involved in cell cycle regulation, including cyclin, cyclin dependent kinase (CDK), CDK-activating kinase (CAK) and CDK inhibitor protein (CKI) [23]. The most obvious success in targeting the cell cycle mechanism is inhibitors of CDK4 and CDK6. With the clinical success of CDK4/6 inhibitors, targeting a specific cyclin could become an effective anticancer strategy [24].

Merghoub et al. found that tomentosin, a natural sesquiterpene lactone extracted from the flowers of *Inula viscosa L.*, can arrest the cell cycle in the G2/M phase [25]. Similarly, Rozenblat et al. found that Tomentosin and Inuviscolide can cause cell cycle arrest at G2/M. This is because these two natural products can inhibit the phosphorylation of CDK1, and the expression levels of Cyclin B1 and CDK1 subsequently decrease [26]. It has also been shown that britannin can prevent the cell transition from the S phase of the cell cycle, thereby reducing the proliferation of acute lymphoblastic leukemia cells, which is achieved by upregulation of p27 and p21 [27]. Rafi et al. isolated and identified two sesquiterpene lactones from the plant *Inula britannica*, O, O-diacetylbritannilactone (OODABL) and O-acetylbritaanilactone (OABL). OODABL and OABL can induce the phosphorylation of Bcl-2 in breast, ovarian, and prostate cancer cell lines and induce G2/M cell cycle arrest [28]. Costunolide, a sesquiterpene lactone derived from *Inula helenium*, can reduce the expression of Cyclin B1 and CDK2 and increase the expression of p21, which leads to cycle arrest in the G2/M phase of leukemia cells [29]. In addition to the previously mentioned, other sesquiterpene lactones such as Bigelovin and Japonicone A (Jap-A) can also arrest the cell cycle in the G0/G1 and S phases, respectively [30, 31]. Bigelovin is a sesquiterpene lactone compound from the plant *Inula helianthus aquatica* and Jap-A is a dimeric sesquiterpene lactone found in the plant *Inula japonica Thunb*.

### Inhibition of tumor metastasis

Malignant tumors are often accompanied by invasion and metastasis, which is also the reason why they are difficult to cure. Effectively inhibiting the invasion and metastasis of tumor cells is the starting point of many clinical and scientific treatments for cancer [32]. It has been found that the matrix metalloproteinase family (MMPs), especially MMP-2 and MMP-9, as common factors promoting invasion and metastasis, play a regulatory role in the development of many tumors [33].

Wang et al. found that ATL could inhibit the metastasis of esophageal cancer through cell experiments and xenograft tumor models in mice. It may act by regulating the Wnt/β-catenin signaling pathway [34]. Bigelovin is cytotoxic to colorectal cancer cells in vitro, reducing their cell viability. Li et al. studied the progression, metastasis and spread of colorectal cancer after Bigelovin treatment by using two colon cancer mouse models, tumor allografts in situ and experimental metastasis models. The results showed that bigelovin significantly inhibited tumor growth and inhibited liver/lung metastasis, possibly by interfering with the IL6/STAT3 and cofilin pathways. Bigelovin has the potential to be developed as an antitumor and antimetastasis agent in colorectal cancer [35]. IATL inhibits breast cancer cell adhesion, migration, and invasion via the p38 MAPK/NF-κB signaling pathway, and the activity and expression of MMP-2 and MMP-9 are downregulated by IATL in a dose-dependent manner [36]. Britanin is a sesquiterpene lactone compound from the plant *Inula japonica*. Britanin reduces lung metastasis. It specifically binds to ZEB1, promotes the degradation of ZEB1 protein, and thus downregulates the protein expression levels of ZEB1, MMP-9 and CD44 [37]. The same results can also be found in another study, Britanin can inhibit the expression of p65 protein and inhibit tumor metastasis [38].

### Induction of autophagy

Autophagy is an evolutionarily conserved intracellular circulatory system and cellular self-degradation process that maintains metabolism and homeostasis. In cancer biology, autophagy plays a dual role in tumor promotion and inhibition and contributes to the development and proliferation of cancer cells. Reduced and abnormal autophagy inhibits the degradation of damaged components or proteins in oxygen-stressed cells, leading to the development of cancer [39]. In recent years, autophagy has been a hot topic in the field of tumor therapy. Inducing autophagy in tumor cells is an effective means to treat cancer [40].

In vitro experiments in one study showed that bigelovin induced the formation of autophagosomes in liver cancer cells. After treatment with bigelovin, LC3B-II and Beclin-1 levels were significantly increased, while p62 levels were decreased. In addition, LC3B-II levels were down-regulated and p62 levels were up-regulated after the addition of autophagy inhibitor 3-MA, indicating that bigelovin-induced autophagy was eliminated by 3-MA by inhibiting the formation of autophagosomes. Moreover, the ability of bigelovin to induce apoptosis was inhibited when 3-MA was added or Beclin-1 was silenced. In the HepG2 xenograft tumor model, LC3B-II level was upregulated in the tumor tissues of the bigelovin administration group, indicating that bigelovin can induce the activation of autophagy in vivo, thereby playing an anti-tumor role [41]. Similarly, ATL has been found to cause the accumulation of autophagosomes in pancreatic cancer cells and can increase LC3B-II levels in a dose—and time-dependent manner [42]. One study has shown that britanin can induce the upregulation of LC3B-II, p62, ATG5 and Beclin-1 and the occurrence of autophagic vacuoles, which triggered autophagy in liver cancer cells. In addition, the upregulation of LC3-II, p62, ATG5, and Beclin1 induced by britanin was reversed when the AMPK inhibitor was added. This suggested that britanin induced autophagy of cells, which was regulated by the activation of AMPK. This phenomenon was also observed in vivo, where p-AMPK and LC3-II levels were upregulated in tumor tissues after administration [43].

### Sensitization Activity

Cancer treatment methods include radiotherapy, chemotherapy, surgery and cellular immunotherapy, which have emerged in recent years. In course of chemotherapy, tumor cells often develop drug resistance, which is one of the most serious obstacles to tumor chemotherapy [44]. A growing number of studies have shown that combining natural products with antitumor drugs can lead to better therapeutic outcomes [45].

Studies have shown that ergolide can enhance the cytotoxicity of vincristine to acute lymphoblastic leukemia cell lines, which indicates the strong synergistic properties of ergolide and vincristine [46]. Another study showed that ATL can induce cell cycle arrest and inhibit cell growth in lung cancer cells. In combination with ATL, the anticancer effect of gemcitabine was significantly enhanced. The authors further demonstrated that ATL can increase ROS levels, thereby inhibiting the AKT/ GSK 3β pathway and ER stress in lung cancer cells. Because of this, treatment with ATL makes lung cancer cells more sensitive to gemcitabine. The combination of ATL and gemcitabine may have potential for clinical use in the treatment of lung cancer [47]. Similarly, other researchers have found that IATL can make colon cancer cells more sensitive to doxorubicin treatment. IATL can lead to ROS accumulation, which leads to activation of the JNK signaling pathway. The synergistic effect of IATL and doxorubicin may be related to this molecular mechanism. Therefore, the combination of IATL and doxorubicin may be a potential treatment for colon cancer [48]. Some researchers extracted 25 sesquiterpene lactones from *Inula japonica*. Among them, compound 24 showed the highest anti-NSLC activity against the paclitaxel- resistant human non-small cell lung cancer cell line A549/PTX and could inhibit cell proliferation and induce cell apoptosis. Compound 24 can significantly inhibit the protein expression of ABCC1, ABCG2 and MDR1, thereby reversing the effect of multidrug resistance and making cells more sensitive to paclitaxel chemotherapy [49].

## Antitumor molecular mechanism of *Inula* sesquiterpene lactones

**Fig. 4:**
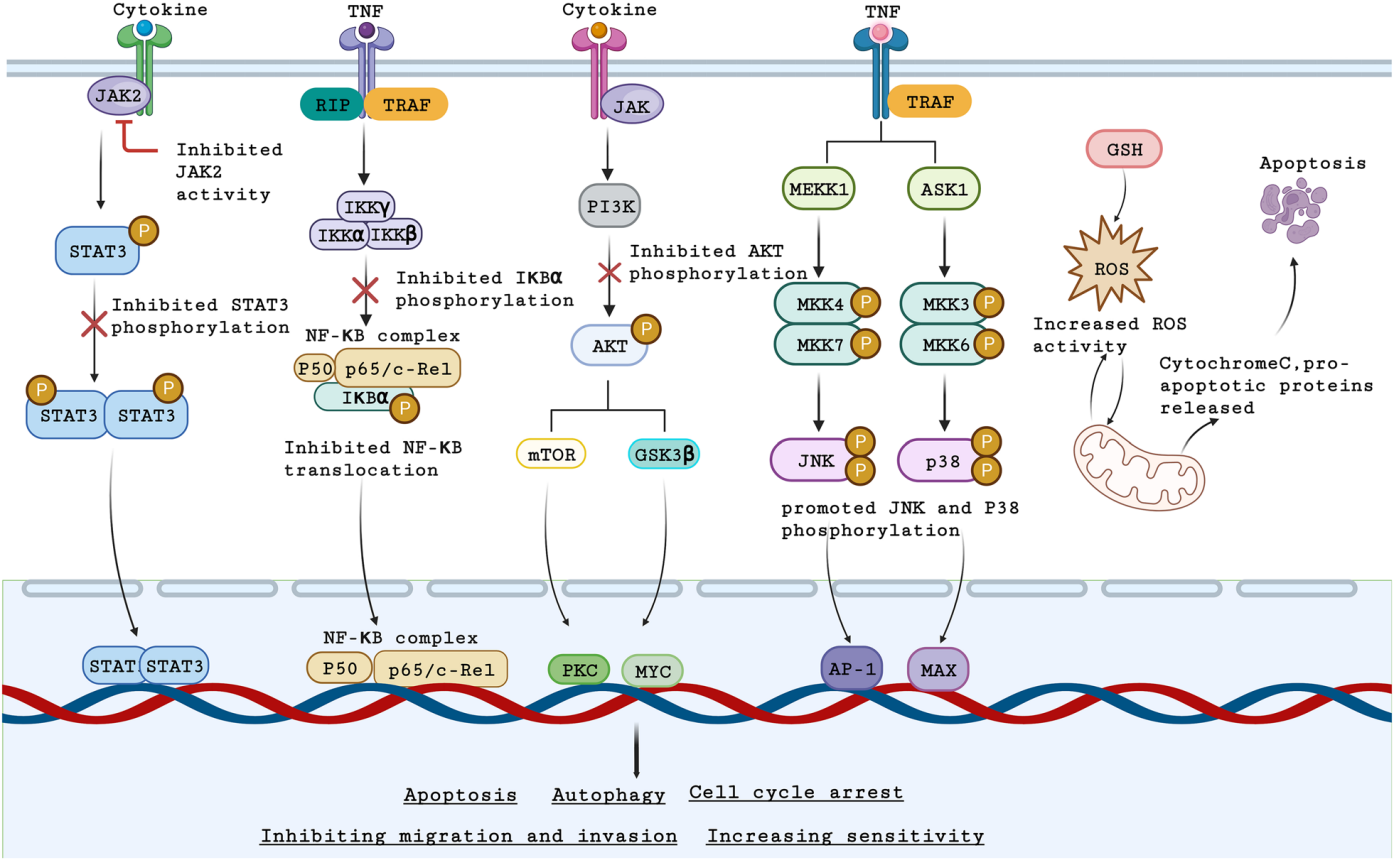
Schematic illustration of the molecular mechanisms underlying the antitumor activity of *Inula* sesquiterpene lactones. (1) *Inula* sesquiterpene lactones inhibit the STAT3 signaling pathway by inhibiting the activity of JAK2 and phosphorylation of STAT3. (2) *Inula* sesquiterpene lactones inhibit the phosphorylation of IκBα and inhibit the activation and nuclear translocation of NF-κB signaling pathway. (3) *Inula* sesquiterpene lactones can directly inhibit PI3K activity and inhibit PI3K/AKT signaling pathway by reducing the expression and phosphorylation of AKT. (4) *Inula* sesquiterpene lactones can stimulate the phosphorylation levels of JNK and p38 and increase their activity to activate the MAPK signaling pathway. (5) *Inula* sesquiterpene lactones can induce ROS production, thus inducing oxidative stress

From the previous review, it can be seen that *Inula* sesquiterpene lactones have various antitumor activities. But how do they exert these antitumor activities? A large number of studies have shown that *Inula* sesquiterpene lactones can achieve antitumor activities by inhibiting NF-kB, STAT3 and PI3K/AKT signaling pathways, activating MAPK signaling pathways and inducing oxidative stress (Table 1 and Fig. 4).

### Inhibition of the NF-κB signaling pathway

The NF-κB pathway is closely associated with cancer. NF-κB plays a key role in the regulation of cytokine-induced gene expression. When the cell is subjected to various intracellular and extracellular stimuli, the IκB kinase is activated, resulting in phosphorylation and ubiquitination of the IκB protein, which is then degraded and the NF-κB dimer is released. The NF-κB dimer is further activated by various post-translational modifications and transferred to the nucleus. In the nucleus, it binds to the target gene to facilitate its transcription [50, 51].

Wang et al. found that IATL can block the p38 MAPK signaling pathway, thereby inhibiting the NF-κB signaling pathway and inhibiting the translocation of NF-κB p65 to the nucleus, resulting in decreased activity of MMP-2 and MMP-9 and inhibiting the invasion and metastasis of breast cancer [36]. Morever, Di et al. discovered a novel mechanism to inhibit the expression of NF-κB p65. The mechanism by which IATL inhibits the expression of NF-κB p65 involves an increased interaction between DR5 and FADD, which is achieved by upregulating DR5, FADD, and cleaved caspase 8. Eventually, ROS-dependent apoptosis occurrs in osteosarcoma cells [52]. ATL can also activate the p38 MAPK pathway and inhibit NF-κB pathway to induce apoptosis of lung cancer and gastric cancer cells, respectively [53, 54]. Yuan et al. analyzed RNA-seq and luciferase reports to show that the NF-κB signaling pathway was significantly inhibited after treatment with bigelovin. Further studies have shown that bigelovin can induce ubiquitination and degradation of IKK-β, and reduce the phosphorylation of IκB-α and p65, leading to downregulation of NF-κB regulatory gene expression [55]. Roozbehani et al. demonstrated that gaillardin exerts its effect by inhibiting the activation of NF-κB, leading to the downregulation of genes regulated by NF-κB, such as COX-2, MMP-9, TWIST-1 and Bcl-2 [56]. One study indicated that britanin can reduce the levels of p65 and phosphorylated p65 and inhibit the NF-κB signaling pathway, increasing the level of downstream molecule IL-2 and a decrease in the level of the IL-10. This suggests that britanin exerts its antitumor effects by enhancing the immune response rather than by promoting apoptosis [57]. Similarly, another study found that britanin inhibits NF-κB activation in pancreatic cancer [58]. One study showed that Japonicone A can inhibit the activity and nuclear translocation of NF-κB induced by TNF-α stimulation and subsequently downregulate genes involved in apoptosis (Bcl-2, Bcl-xl, TRAF2) and cell cycle-related genes (Cyclin D, MYC). Therefore, Japonicone A can inhibit the growth of lymphatic cancer in vivo and in vitro [59]. Ergolide is a sesquiterpene lactone derived from *Inula britannica*. Chun et al. demonstrated that ergolide inhibits NF-κB-dependent gene transcription in HeLa cells stimulated by z12-O-tetradecanoylphorbol 13acetate (TPA) due to inhibition of NF-κB DNA binding activity and nuclear translocation of NF-κB p65 subunits [60]. In addition, ergolide has been shown to significantly inhibit the NF-κB signaling pathway in Jurkat T cells [61].

### Inhibition of the STAT3 signaling pathway

STAT3 was first discovered as an oncogene, that is involved in various physiological pathways, such as cell growth, differentiation and apoptosis. Phosphorylated STAT3 rapidly enters the nucleus, forms homodimers or heterodimers from monomers, acts as a transcription factor, binds to promoters of target genes, and activates transcription. Under the stimulation of carcinogenic signals, STAT3 is continuously activated to remain in the nucleus in an activated state, continuously activating target genes, and promoting the growth of tumor cells [62].

One study extracted the hexane fraction of *Inula helenium L* (HFIH), including alantolactone, isoalantolactone, igalan, dugesialactone, and alloantolactone. The results showed that HFIH could selectively inhibit the phosphorylation of STAT3 at tyrosine 705 and thus significantly inhibit the activation of STAT3. HFIH can also downregulate the expression of STAT3 target genes, such as Cyclin D1, MYC and Bcl-2, and induce apoptosis mediated by caspase. The researchers also conducted in vivo experiments, which also confirmed this conclusion [63]. Ahmad et al. found that ATL can reduce cell viability and induce apoptosis. This is because ATL can inhibit the expression of STAT3 and survival proteins [64]. Bigelovin potently inhibits STAT3 signaling by inactivating JAK2 and induces apoptosis of a variety of human cancer cells in vitro [65].

### Activation of the MAPK signaling pathway

The MAPK signaling pathway is an important pathway in the eukaryotic signal transmission network. It plays a key role in gene expression regulation and cytoplasmic functional activities. Five different MAPK signaling pathways have been identified in mammalian organisms. The ERK1/2 signal transduction pathway regulates cell growth and differentiation, and the JNK and p38 MAPK signal transduction pathways play important roles in stress responses such as inflammation and apoptosis. Abnormalities in MAPK signaling pathways have been shown to be associated with various types of cancer [66].

It has been found that IATL can induce apoptosis of human hepatocellular carcinoma Hep3B cells by activating the MAPK signaling pathway. After treatment with IATL, levels of p-ERK and p-JNK increased without any change in their total proteins. When treated with potent JNK inhibitors, the anticancer effects of IATL are significantly reduced [67]. Another study has also shown that IATL strongly induced p-JNK expression. The cell death induced by IATL was also significantly reversed when treated with specific JNK inhibitors. When IATL combined with doxorubicin, JNK phosphorylation levels increased significantly. This suggested that IATL plays an antitumor role by activating JNK signaling pathway and increases the toxicity of doxorubicin to colorectal cancer cells [48]. 1,6-O, O-diacetylbritannilactone (OODBL) is a sesquiterpene lactone isolated from *Inula Britannica*. It was found that the activation of the MAPK and JNK signaling pathways may play an important role in OODBL-induced apoptosis [68].

### Inhibition of the PI3K/AKT signaling pathway

The PI3K/AKT signaling pathway plays an important role in regulating a variety of biological responses, including metabolism, cell survival and growth. AKT is activated by PI3K. Upon activation, AKT targets several downstream molecules, altering molecular activity by phosphorylation or by forming complexes [69].

ATL can enhance the anticancer effects of gemcitabine through ROS-mediated activation of the AKT/GSK3β pathway [47]. In addition, ATL can exert anti-liver cancer activity. When treated with ATL, the phosphorylation levels of AKT was decreased. Further studies have shown that ATL can induce apoptosis through ROS-mediated AKT signaling inhibition and PINK1-mediated mitochondrial autophagy [70]. Igalan is one of the sesquiterpene lactones found in *Inula helenium* c, which can increase the inactive form of GSK3β and the phosphorylated form of AKT [71]. Costunolide, a sesquiterpene lactone extracted from *Inula helenium* L., has antiproliferation effects on several tumor cells [72]. Costunolide can significantly enhance the anti-proliferative activity of doxorubicin against drug-resistant cell lines by inhibiting the PI3K/AKT pathway and downregulating the expression of P-glycoprotein [73]. One study showed that eupatolide, the sesquiterpene lactone isolated from the medicinal plant *Inula britannica*, can sensitize human breast cancer cells to TRAIL-induced apoptosis by downregulating the expression of cellular FLICE inhibitory protein (c-FLIP) through the inhibition of AKT phosphorylation. Euaptolide can inhibit AKT phosphorylation in a dose- and time-dependent manner [74]. Tomentosin is the most representative sesquiterpene lactone extracted by *I. viscosa*. which can inhibit cell proliferation and induce apoptosis through the inhibition of the mTOR/PI3K/ AKT signaling pathway [75].

### Induction of oxidative stress

ROS is the products of normal aerobic metabolism in the body and are a general term for a class of substances composed of oxygen that are active in nature. ROS plays an important role in the maintenance of the cell cycle, gene expression and environmental homeostasis in the body [76]. Oxidative stress refers to the excessive production of highly active molecules such as ROS in the body, the degree of oxidation exceeding the removal of oxides, and the imbalance between the oxidation system and the antioxidant system, resulting in tissue damage [77]. To resist these adverse effects, the body has developed a complex oxidative stress response system to mitigate damage to cells. NRF2, as a key transcription factor regulating antioxidant stress, plays an important role in inducing the body's antioxidant response [78]. Small molecule drugs from different sources, including natural small molecule drugs and extracts of traditional Chinese medicine, are inducers or scavengers of reactive oxygen species, which bring new ideas for artificial intervention of intracellular ROS levels.

Some studies have shown that sesquiterpenoids can induce oxidative stress and affect tumor progression. ATL can induce ROS production and activate the p38 MAPK pathway by inhibiting TrxR activity, leading to apoptosis of gastric cancer cells. However, this effect can be reversed when treated with the ROS scavenger. ATL can be used in combination with glutathione inhibitors to synergistically exert antitumor effects [79]. Similarly, it has also been found that the α-methylene-γ-lactone part of ATL and the Sec residue in TrxR are essential for ATL to target TrxR. The study found that the level of TrxR in HeLa cells was significantly increased after treatment with ATL, suggesting that ATL induces ROS accumulation and ultimately induces cell apoptosis [80]. Moreover, ATL can increase ROS levels and the accumulation of cellular oxidized guanine (8-oxoG), resulting in oxidative DNA damage. Therefore, the cell cycle is blocked in G1 phase and apoptosis is significantly induced [81]. ATL can enhance ROS-induced LATS kinase activity, thereby increasing YAP1/ TAZ phosphorylation. Therefore, ATL can target the ROS-YAP pathway to inhibit tumor cell growth [82]. Moreover, similar findings were confirmed in B-cell acute lymphoblastic leukemia cells and lung cancer cells [83, 84]. 2-α-Hdroxyeudesma-4,11(13)-dien-8β,12-olide (HEDO), extracted from *Inula britannica*, upregulates intracellular ROS and increases the depolarization of mitochondrial membrane potential, leading to cell cycle arrest and apoptosis [85]. In addition, IATL has been shown to induce the activity of phase 2 enzyme by stimulating the accumulation of NRF2 in the nucleus. In this process, the PI3K/AKT/NRF2 signaling pathway may be partially involved in the nuclear translocation of NRF2 [86]. Similarly, Chen et al. found that ATL can also inhibit esophageal adenocarcinoma cells by inhibiting NRF2 to increase ROS. Knocking down NRF2 enhanced the apoptosis-inducing effect of ATL, while overexpression of NRF2 reduced the apoptosis-inducing effect of ATL. The same results were found in vivo [87].

## Molecular targets of *Inula* sesquiterpene lactones

In recent years, with the rise of the concept of precision diagnosis and treatment, new antitumor drugs, represented by small molecule targeted drugs and large molecule monoclonal antibodies, have emerged rapidly. Compared with the traditional anti-chemotherapy drugs, the new anti-tumor drugs have high specificity and less toxic side effects and have a significant effect on a variety of malignant tumors. Therefore, the discovery and confirmation of drug molecular targets is of great significance for the research and development of innovative drugs. According to the difference of the mechanism of target action, the molecular targets of tumor action can be divided into targeting the regulatory mechanism of tumor formation, targeting the tumor microenvironment, tumor immunotherapy, tumor markers and targeting tumor stem cells. As shown in Table 1, several researchers have studied the molecular targets of *Inula* sesquiterpene lactones. These molecular targets will be discussed in turn.

### Binding to TNF‑α

TNF is a pro-inflammatory cytokine secreted mainly by mononuclear macrophages. It is one of the most important cytokines in the tumor microenvironment and has the strongest antitumor effect known to date [5]. JAP-A can directly bind to TNF-α, thereby inhibiting its binding to TNFR1. Its direct binding to cytokines leads to the blocking of downstream signaling events, particularly the activation of NF-κB. The results of in vivo experiments showed that Jap-A protected mice against TNF-α / D-galactosamine-induced acute hepatitis but did not affect host antiviral immunity in adenovirus-infected mice [88]. Inflammation and persistent infection may lead to various human malignancies, so this study has a good reference for the antitumor effect of Jap-A. In addition, Bailly et al. constructed a molecular model of the compound/target interaction. Molecular docking showed that the compound can be used as an interfacial ligand, which fits well at the junction between two TNF-α subunits and binds to proteins through a series of molecular interactions such as hydrogen bonding and van der Waals contacts [89].

### Binding to MDM2, UbcH5c and Keap1

Ubiquitination (Ub) is a process by which ubiquitin molecules classify intracellular proteins, select target protein molecules, and modify target proteins specifically under the action of a series of special enzymes. A series of enzymes like ubiquitin-activating enzyme (E1), ubiquitin-conjugating enzymes (E2), ubiquitin ligase (E3), and deubiquitinating enzymes (DUBs) are involved in ubiquitin signaling, controlling protein post-translational ubiquitination and regulating protein stability [90].

UbcH5c is an E2 ubiquitin-conjugative enzyme. A Study have shown that UbcH5c is overexpressed in pancreatic cancer and associated with poor prognosis of pancreatic cancer [91]. DHPO, one *Inula* sesquiterpene lactone, can directly bind to UbcH5c by forming hydrogen bonds with the amino groups of Leucine 86 and Arginine 90 and inhibit its function, thereby inhibiting the NF-κB signaling pathway. It exerts anti-tumor effects in vivo and in vitro and provides a candidate drug for the treatment of pancreatic cancer [91]. In 2014, the team first discovered and reported a natural compound IJ-5, a new sesquiterpene lactone component found from *I. japonica Thunb*, which specifically binds to UbcH5 [92]. The researchers optimized the structure of IJ-5 to develop a new generation of more specific UbcH5c inhibitor compound called compound 6d which may be used to treat inflammatory and autoimmune diseases [93].

MDM2 is an E3 ubiquitin ligase that is frequently overexpressed in cancer cells. p53 is a key tumor suppressor gene in human cells, and some oncogenes, such as MDM2 can directly bind to p53 protein to form p53-MDM2 complex, which can inhibit p53-mediated transcriptional activation. Therefore, p53-MDM2 interaction has become an important drug target for anticancer drugs [94]. Qin et al. found that Jap-A could directly bind to MDM2, block MDM2-p53 interaction, promote MDM2 ubiquitination and proteasomal degradation and inhibit MDM2 gene transcription. In addition, the expression level of MDM2 was significantly decreased in the presence of Jap-A in the mouse breast cancer MDA-MB-231 model. Jap-A can competitively bind to the hydrophobic pockets of MDM2, thus preventing critical p53 residues from binding to them. The ability of JapA to bind MDM2 protein was higher than that of p53 residue [95]. Bailly et al. further showed that the compound may fit between two α-helices and interaction with the protein via H-bonds with residues Lysine 51 and Glutamine 24, and via several other molecular contacts [89].

Keap1 is a Cullin3(Cul3) -dependent E3 ubiquitin ligase complex substrate adaptor protein, which can assemble with Cullin3 and Rbx1 to form a functional E3 ubiquitin ligase complex (Keap1-Cul3-E3) to regulate NRF2. Keap1 contains three functional domains, including a BTB domain, an IVR domain and a Kelch or DGR domain. The BTB domain binds Cul3 and is required for Keap1 dimerization. The BTB domain binds Cul3, which is required for Keap1 dimerization. The team led by Zhang discovered that britanin directly binds to the cysteine residue (Cys-151) within the BTB domain of Keap1, thereby disrupting Keap1's role as an adapter for the Keap1-Cullin3 ubiquitin ligase complex, ultimately resulting in activation of the NRF2 protective pathway [96]. Under physiological conditions, NRF2 plays a crucial role in maintaining cellular reduction–oxidation (REDOX) homeostasis and exerts potent anti-inflammatory functions as well as additional anti-cancer activities, thereby supporting cell survival. Hence, the activation of NRF2 is pivotal for cancer chemoprevention [97]. However, it should be noted that NRF2 has a dual role in cancer. Excessive activation of NRF2 can confer various advantages to cancer cells, including protection against apoptosis and senescence, promotion of cancer cell metastasis, and development of resistance to chemotherapy and radiotherapy [98]. Therefore, further investigation is warranted to elucidate the mechanism by which britanin exerts its anti-tumor effect through targeting Keap1.

### Binding to NLRP3

Inflammatory cytokines mediated by NOD-like receptor thermal protein domain associated protein 3 (NLRP3) inflammasomes play a dual role in mediating human diseases. Although they are deleterious in the pathogenesis of inflammatory and metabolic diseases, they have beneficial effects in many infectious diseases and some cancers. Therefore, fine-tuning NLRP3 inflammasome activity is essential to maintain proper cellular homeostasis and health [99]. Ergolide is a potent inhibitor of NLRP3-mediated pyroptosis in vitro and in vivo. Furthermore, we confirmed that ergolide irreversibly binds to the NLRP3 NACHT domain to prevent assembly and activation of the NLRP3 inflammasome [100]. Inflammation and persistent infection may lead to various human malignancies. Now there are data showing that NLRP3 inflammasome polymorphism is associated with different malignant tumors such as gastric cancer, colon cancer and lung cancer [101]. Therefore, this study is of great significance for the study of anti-tumor molecular targets of ergolide.

### Binding to protein kinas JAK2 and PLK1

Polo-like kinase-1 (PLK1), a serine/threonine protein kinase involved in the initiation, maintenance and termination of mitosis, is highly expressed in a variety of cancers. [102]. Racemolactone I is a new sesquiterpene lactone isolated from *Inula racemosa*. One study performed molecular docking and simulation studies to confirm that racemolactone I binds to the PLK-1 active sites to form a stable complex. The residues that racemolactone I interacted with PLK-1 were mainly Leucine 59, Glycine 60, Lysine 61, Glycine 62, etc. [102, 103].

The protein encoded by the JAK2 gene is a non-receptor tyrosine kinase, a member of the Janus kinase family. JAK/STAT is a very important signaling pathway. Many cytokines and growth factors can induce cell proliferation, differentiation and apoptosis through this signaling pathway [104]. Zhang et al. examined the effects of bigelovin on JAK2 enzymatic activity in vitro and demonstrated that bigelovin can inactivate its enzymatic activity. These data strongly suggested that bigelovin can inhibit the JAK2/STAT3 signaling pathway by directly inactivating JAK2. The results of the LC–MS analysis suggested that biglovin may react with the cysteine residues of JAK2, resulting in the inactivation of JAK2 [65].

### Binding to PD-L1

In recent years, immunology oncology therapy has become one of the important methods for the treatment of advanced malignant tumors. Immunology oncology therapy does not directly attack cancer cells but fights tumors by activating the body's own immune system, which has good safety and tolerance [105]. Programmed death 1 (PD-1), a member of the CD28 superfamily, is an important immunosuppressive molecule, and its Ligand is programmed cell death-ligand 1 (PD-L1) [106]. PD-1/PD-L1 inhibitors can combine with PD-1 or PD-L1 to block the inhibitory effect of tumor cells on immune function, restore the activity of T cells, and enhance the immune response. At present, the approved PD-1/PD-L1 inhibitors are macromolecular antibody drugs. Monoclonal antibodies have many disadvantages, including poor oral bioavailability, poor membrane permeability, and difficulties in transportation and storage [107]. In order to avoid these shortcomings, more and more researchers are exploring small-molecule chemicals as PD-1/PD-L1 inhibitors. These small molecule inhibitors are currently in various stages of preclinical or clinical research. Zhang et al. found that britanin had a significant inhibitory effect on the protein and mRNA levels of PD-L1 in HCT116, A549, HeLa and Hep3B cell lines. Britannin can inhibit PD-L1 to enhance the activity of cytotoxic T lymphocytes and inhibit tumor cell proliferation and angiogenesis. By using molecular docking assay, they proposed that britanin can bound to the PD-L1 binding pocket components of Asparagine 131, Alanine 132, Glutamate 72, and Lysine 89. In vivo and in vitro experiments have also shown that britanin can suppress PD-L1 expression by blocking the interaction between HIF-1α and MYC. This study provides a reference for the development of natural small molecule inhibitors of PD-L1 [108].

## Summary and prospects

Studies have shown that *Inula* sesquiterpene lactones hold promise as potential antitumor drugs, demonstrating significant inhibitory effects on gastric cancer, breast cancer, cervical cancer, colon cancer, and other types of tumors. However, current research on *Inula* sesquiterpene lactones still faces certain limitations. Firstly, most of the studies investigating the antitumor mechanism have been conducted in vitro, with relatively limited in vivo experiments. To date, no *Inula* sesquiterpene lactones have been approved by the Food and Drug Administration (FDA) for clinical trials. However, other sesquiterpene lactones, such as mipsagargin, which was synthesized on basis of natural product thapsigargin, have been in clinical research. One phase II multicenter, single-arm study was designed to evaluate the safety and efficacy of mipsagargin in adult patients with advanced hepatocellular carcinoma (HCC) who had progressed on or after treatment with sorafenib or were intolerant of sorafenib. The results showed that the regimen was well tolerated, stabilized the disease, and prolonged time to disease progression (TTP) in patients previously treated with sorafenib. This suggests that mipsagargin may have clinical activity in HCC, including in patients with advanced refractory HCC [109]. Therefore, further studies involving more animal experiments are needed to verify the antitumor mechanism and evaluate effectiveness and safety so as to establish a solid foundation for future clinical trials. Secondly, there is a scarcity of studies focusing on the specific molecular targets of *Inula* sesquiterpene lactones, with most investigations being limited to certain pathways. In modern drug discovery, it is crucial to identify the specific molecular target in order to understand the mechanism of action of the drug, assess potential toxicity, and overcome possible resistance mechanisms. Therefore, researchers should conduct more experiments to explore the molecular targets of *Inula* sesquiterpene lactones. Techniques such as RNA-seq [110], proteomics [111], CETSA [112], SPR [113] and others can be employed to identify and verify the binding ability with these targets. Last but not least, the relationship between the structure of monomer compounds and their biological activity remains inadequately explored. The development story of ZD03 may provide us with some insights. Professor Weidong Zhang 's research group at the Naval Medical University discovered the britanin as a lead compound, which initially demonstrated anti-inflammatory activity [96]. Subsequent assessments of its pharmacokinetic properties prompted the development of the salt form ZD03, leading to significant improvements in solubility, bioavailability, and metabolic stability compared to the original lead compound. As a result, ZD03 successfully advanced to clinical studies. To advance drug development, it is crucial for researchers to further elucidate the correlation between structure and drug efficacy. Purposefully modifying the chemical structure of *Inula* sesquiterpene lactones can yield lead compounds that are more effective and less toxic, ultimately suitable for clinical use. By elucidating this relationship, researchers can pave the way for the creation of novel drugs that offer enhanced therapeutic benefits. At present, some people have modified the structure of *Inula* sesquiterpene lactones. 1-O-acetyl-6-O-lauroylbritannilactone (ABL-L) is a semi-synthetic analogue of the natural product 1-O-acetylbrominolactone (ABL). One study has found that the inhibitory effect of ABL-L on tumor cell lines is 4–10 times higher than that of ABL. The Further study has found that ABL-L has a good anti-cancer effect on human laryngocarcinoma cells, which can induce cell apoptosis and block the cell cycle in G1 phase [114]. Therefore, ABL-L may be a potential treatment for laryngocarcinoma. ABL-N, another derivative of ABL, was also synthesized and studied. The study has shown that ABL-N can induce apoptosis of breast cancer cells in vitro, inhibit cell proliferation and significantly inhibit tumor growth in vivo. This may work by activating the MAPK signaling pathway [115]. Therefore, ABL-N may be a potential drug for breast cancer prevention and intervention. Studies on the Inula sesquiterpene lactones have further demonstrated their potential as antitumor agents, but the research in this area is still lacking and more researchers are needed to participate.

At present, the problem of drug resistance has made the treatment of tumors into a dilemma. It has been found that the *Inula* sesquiterpene lactones can be combined with doxorubicin and other chemotherapy drugs to increase their sensitivity and reverse multi-drug resistance. More importantly, studies have shown that they can also be combined with anti-PD-1 antibodies to significantly increase the proportion of CD8 T cells. In addition, combination therapy enhanced anti-tumor immunity by reducing the number of myeloid suppressor cells and increasing the number of M1-like macrophages [116]. These results indicate that the *Inula* sesquiterpene lactones have great value in chemotherapy and immunotherapy. Therefore, research on them may provide help for future cancer prevention and treatment. They may be used as excellent anti-cancer drugs in clinical treatment, and they may be combined with chemotherapy drugs or immunotherapy drugs to prolong the survival of patients and improve their survival rates.

In summary, the structure, structure–activity relationship, anti-tumor activity, mechanism of action and molecular targets of *Inula* sesquiterpene lactones were reviewed in this paper. They have anti-tumor activities such as promoting cell apoptosis and inducing cell cycle arrest. These activities mainly play a role by regulating NF-κB, STAT3 and other signaling pathways or inducing oxidative stress. The limitations of the present study and the application value of *Inula* sesquiterpene lactones were also discussed in this paper, which can provide reference for further study of their anti-tumor effects.

## Data Availability

No data was used for the research described in the article.

## Abbreviations

NF-κB: Nuclear factor-κB
STAT3: Signal transducer and activator of transcription 3
PI3K: Phosphatidylinositol-3-kinase
MAPK: Mitogen-activated protein kinase
TNF-α: Tumor necrosis factor-alpha
MDM2: Mouse double minute 2
ZEB1: Zinc finger E-box-binding homeobox1
TFEB: Transcription factor EB
JAK2: Janus kinase 2
PLK1: Polo-like kinase-1
ERS: Endoplasmic reticulum stress
DR: Death receptor
TRAIL: TNF-related apoptosis-inducing ligand
CDK: Cyclin dependent kinase
CAK: CDK-activating kinase
CKI: CDK inhibitor protein
MMPs: Matrix metalloproteinase family
LC3B-II: Microtubule-associated light chains 3B-II
CTSB/CTSD: Cathepsin D /cathepsin D
ATG5: Autophagy-associated 5
ROS: Reactive oxygen species
GSK 3β: Glycogen synthase kinase 3β
JNK: C-Jun N-terminal kinase
EMT: Epithelial-mesenchymal transition
SMAD3: Mothers against decapentaplegic homolog 3
FADD: Fas-associating protein with a novel death domain
IKK-β: Kappa-B kinase-beta; IL-2, interleukin-2
TPA: Z12-O-tetradecanoylphorbol 13acetate
PINK1: PTEN-induced putative kinase 1
c-FLIP: Cellular FLICE inhibitory protein
NRF2: Nuclear factor erythroid2-related factor 2
TrxR: Thioredoxin reductase
LATS: Large tumor suppressor
YAP1: Yes-associated protein 1
TAZ: PDZ-binding motif
TNFR1: TNF receptor 1
DUBs: Deubiquitinating enzymes
Keap1: Kelch-like ECH-associated protein 1
NLRP3: NOD-like receptor thermal protein domain associated protein 3
PD-1: Programmed death 1
PD-L1: Programmed cell death-ligand 1
CETSA: Cellular thermal shift Assay
SPR: Surface plasmon resonance
Bcl-2: B-cell lymphoma-2
Bax: Bcl-2-associated X
AMPK: Adenosine 5′-monophosphate (AMP)-activated protein kinase
mTOR: Mammalian target of rapamycin
HIF-1α: Hypoxia-inducible factor-1α
MYC: Cellular-myelocytomatosis viral oncogene
ABCG2: Adenosine triphosphate (ATP)-binding cassette transporter G2
ABCC1: ATP binding cassette subfamily C member 1
MDR1: Multidrug Resistance Protein 1
IκB: Inhibitor kappa B
COX-2: Cyclooxygenase-2
ERK1/2: Extracellular regulated protein kinases ½
DARTS: Drug affinity-responsive target stabilization
MAPT: Microtubule-Associated Protein Tau
REDOX: Reduction–oxidation
TRAF2: TNF receptor associated factor 2
RIP: Ribosome-inactivating protein
ASK1: Apoptosis signal-rgulating kinase 1
PKC: Protein kinase C
GSH: Glutathione
MAX: Myc associated factor X
AP-1: Activator protein 1
MEKK: Mitogen-activated protein kinase kinase kinase
MKK: Mitogen-activated protein Kinase Kinase
Iκκ: Inhibitor of kappa B kinase

## References

[1] Guo M, Jin J, Zhao D, Rong Z, Cao LQ, Li AH (2022). Research advances on anti-cancer natural products. Front Immunol.

[2] Wang G-W, Qin J-J, Cheng X-R, Shen Y-H, Shan L, Jin H-Z (2014). Inula sesquiterpenoids: structural diversity, cytotoxicity and anti-tumor activity. Expert Opin Investig Drugs.

[3] Sun C, Jia Z, Huo X, Tian X, Feng L, Wang C (2021). Medicinal *Inula* species: phytochemistry, biosynthesis, and bioactivities. Am Chin Med.

[4] Yang L, Wang X, Hou A, Zhang J, Wang S, Man W (2021). A review of the botany, traditional uses, phytochemistry, and pharmacology of the Flos *Inulae*. J Ethnopharmacol.

[5] Balkwill F (2009). Tumour necrosis factor and cancer. Nat Rev Cancer.

[6] Firestone GL, Sundar SN (2009). Anticancer activities of artemisinin and its bioactive derivatives. Expert Rev Mol Med.

[7] Mathema VB, Koh Y-S, Thakuri BC, Sillanpää M (2011). Parthenolide, a sesquiterpene lactone, expresses multiple anti-cancer and anti-inflammatory activities. Inflammation.

[8] Jaskulska A, Janecka AE, Gach-Janczak K (2020). Thapsigargin—from traditional medicine to anticancer drug. Int J Mol Sci.

[9] Cheikh IA, El-Baba C, Youssef A, Saliba NA, Ghantous A, Darwiche N (2022). Lessons learned from the discovery and development of the sesquiterpene lactones in cancer therapy and prevention. Expert Opin Investig Drugs.

[10] Li Q, Wang Z, Xie Y, Hu H (2020). Antitumor activity and mechanism of costunolide and dehydrocostus lactone: two natural sesquiterpene lactones from the Asteraceae family. Biomed Pharmacother.

[11] Li Y, Ni Z-Y, Zhu M-C, Dong M, Wang S-M, Shi Q-W (2012). Antitumour activities of sesquiterpene lactones from *Inula helenium* and *Inula japonica*. Zeitschrift für Naturforschung C.

[12] Konishi T, Shimada Y, Nagao T, Okabe H, Konoshima T (2002). Antiproliferative sesquiterpene lactones from the roots of *Inula helenium*. Biol Pharm Bull.

[13] Dong S, Tang J-J, Zhang C-C, Tian J-M, Guo J-T, Zhang Q (2014). Semisynthesis and in vitro cytotoxic evaluation of new analogues of 1-O-acetylbritannilactone, a sesquiterpene from *Inula britannica*. Eur J Med Chem.

[14] Pistritto G, Trisciuoglio D, Ceci C, Garufi A, D'Orazi G (2016). Apoptosis as anticancer mechanism: function and dysfunction of its modulators and targeted therapeutic strategies. Aging.

[15] Carneiro BA, El-Deiry WS (2020). Targeting apoptosis in cancer therapy. Nat Rev Clin Oncol.

[16] Oh Y-T, Sun S-Y (2021). Regulation of cancer metastasis by trail/death receptor signaling. Biomolecules.

[17] Fallahian F, Aghaei M, Abdolmohammadi MH, Hamzeloo-Moghadam M (2016). Molecular mechanism of apoptosis induction by Gaillardin, a sesquiterpene lactone, in breast cancer cell lines. Cell Biol Toxicol.

[18] Cui L, Bu W, Song J, Feng L, Xu T, Liu D (2017). Apoptosis induction by alantolactone in breast cancer MDA-MB-231 cells through reactive oxygen species-mediated mitochondrion-dependent pathway. Arch Pharm Res.

[19] Chen C-N, Huang H-H, Wu C-L, Lin CPC, Hsu JTA, Hsieh H-P (2007). Isocostunolide, a sesquiterpene lactone, induces mitochondrial membrane depolarization and caspase-dependent apoptosis in human melanoma cells. Cancer Lett.

[20] Wang Q, Gao S, Wu G, Yang N, Zu X, Li W (2018). Total sesquiterpene lactones isolated from *Inula helenium* L. attenuates 2, 4-dinitrochlorobenzene-induced atopic dermatitis-like skin lesions in mice. Phytomedicine.

[21] Xu L, Sun Y, Cai Q, Wang M, Wang X, Wang S (2023). Research progress on pharmacological effects of isoalantolactone. J Pharm Pharmacol.

[22] Lu Z, Zhang G, Zhang Y, Hua P, Fang M, Wu M (2018). Isoalantolactone induces apoptosis through reactive oxygen species-dependent upregulation of death receptor 5 in human esophageal cancer cells. Toxicol Appl Pharmacol.

[23] Peyressatre M, Prével C, Pellerano M, Morris M (2015). Targeting cyclin-dependent kinases in human cancers: from small molecules to peptide inhibitors. Cancers.

[24] Suski JM, Braun M, Strmiska V, Sicinski P (2021). Targeting cell-cycle machinery in cancer. Cancer Cell.

[25] Merghoub N, El Btaouri H, Benbacer L, Gmouh S, Trentesaux C, Brassart B (2017). Tomentosin induces telomere shortening and caspase-dependant apoptosis in cervical cancer cells. J Cell Biochem.

[26] Rozenblat S, Grossman S, Bergman M, Gottlieb H, Cohen Y, Dovrat S (2008). Induction of G2/M arrest and apoptosis by sesquiterpene lactones in human melanoma cell lines. Biochem Pharmacol.

[27] Mohammadlou H, Hamzeloo-Moghadam M, Yami A, Feizi F, Moeinifard M, Gharehbaghian A (2021). Britannin a sesquiterpene lactone from inula aucheriana exerted an anti-leukemic effect in acute lymphoblastic leukemia (ALL) cells and enhanced the sensitivity of the cells to vincristine. Nutr Cancer.

[28] Rafi MM, Bai N-S, Ho C-T, ROSEN RT, WHITE E, PEREZ D, (2005). A sesquiterpenelactone from *Inula britannica* induces anti-tumor effects dependent on Bcl-2 phosphorylation. Anticancer Res.

[29] Cai H, He X, Yang C (2018). Costunolide promotes imatinib-induced apoptosis in chronic myeloid leukemia cells via the Bcr/Abl-Stat5 pathway. Phytother Res.

[30] Zeng G-Z, Tan N-H, Ji C-J, Fan J-T, Huang H-Q, Han H-J (2009). Apoptosis inducement of bigelovin from *Inula helianthus-aquatica* on human Leukemia U937 cells. Phytother Res.

[31] Du Y, Gong J, Tian X, Yan X, Guo T, Huang M (2015). Japonicone A inhibits the growth of non-small cell lung cancer cells via mitochondria-mediated pathways. Tumor Biol.

[32] Steeg PS (2006). Tumor metastasis: mechanistic insights and clinical challenges. Nat Med.

[33] John A, Tuszynski G (2001). The role of matrix metalloproteinases in tumor angiogenesis and tumor metastasis. Pathol Oncol Res.

[34] Wang Z, Hu Q, Chen H, Shi L, He M, Liu H (2021). Inhibition of growth of esophageal cancer by alantolactone via Wnt/β-catenin signaling. Anticancer Agents Med Chem.

[35] Li M, Yue GG-L, Song L-H, Huang M-B, Lee JK-M, Tsui SK-W (2018). Natural small molecule bigelovin suppresses orthotopic colorectal tumor growth and inhibits colorectal cancer metastasis via IL6/STAT3 pathway. Biochem Pharmacol.

[36] Wang J, Cui L, Feng L, Zhang Z, Song J, Liu D (2016). Isoalantolactone inhibits the migration and invasion of human breast cancer MDA-MB-231 cells via suppression of the p38 MAPK/NF-κB signaling pathway. Oncol Rep.

[37] Lu H, Wu Z, Wang Y, Zhao D, Zhang B, Hong M (2022). Study on inhibition of Britannin on triple-negative breast carcinoma through degrading ZEB1 proteins. Phytomedicine.

[38] Li H, Du G, Yang L, Pang L, Zhan Y (2020). The antitumor effects of britanin on hepatocellular carcinoma cells and its real-time evaluation by in vivo bioluminescence imaging. Anticancer Agents Med Chem.

[39] Bhat P, Kriel J, Shubha Priya B, Basappa SNS, Loos B (2018). Modulating autophagy in cancer therapy: advancements and challenges for cancer cell death sensitization. Biochem Pharmacol.

[40] Kocaturk NM, Akkoc Y, Kig C, Bayraktar O, Gozuacik D, Kutlu O (2019). Autophagy as a molecular target for cancer treatment. Eur J Pharm Sci.

[41] Wang B, Zhou T-Y, Nie C-H, Wan D-L, Zheng S-S (2018). Bigelovin, a sesquiterpene lactone, suppresses tumor growth through inducing apoptosis and autophagy via the inhibition of mTOR pathway regulated by ROS generation in liver cancer. Biochem Biophys Res Commun.

[42] He R, Shi X, Zhou M, Zhao Y, Pan S, Zhao C (2018). Alantolactone induces apoptosis and improves chemosensitivity of pancreatic cancer cells by impairment of autophagy-lysosome pathway via targeting TFEB. Toxicol Appl Pharmacol.

[43] Cui Y-Q, Liu Y-J, Zhang F (2018). The suppressive effects of Britannin (Bri) on human liver cancer through inducing apoptosis and autophagy via AMPK activation regulated by ROS. Biochem Biophys Res Commun.

[44] Wu Q, Yang Z, Nie Y, Shi Y, Fan D (2014). Multi-drug resistance in cancer chemotherapeutics: Mechanisms and lab approaches. Cancer Lett.

[45] Yuan R, Hou Y, Sun W, Yu J, Liu X, Niu Y (2017). Natural products to prevent drug resistance in cancer chemotherapy: a review. Ann N Y Acad Sci.

[46] Yami A, Hamzeloo-Moghadam M, Darbandi A, Karami A, Mashati P, Takhviji V (2020). Ergolide, a potent sesquiterpene lactone induces cell cycle arrest along with ROS-dependent apoptosis and potentiates vincristine cytotoxicity in ALL cell lines. J Ethnopharmacol.

[47] Wang J, Zhang Y, Liu X, Wang J, Li B, Liu Y (2019). Alantolactone enhances gemcitabine sensitivity of lung cancer cells through the reactive oxygen species-mediated endoplasmic reticulum stress and Akt/GSK3β pathway. Int J Mol Med.

[48] Wu F, Shao R, Zheng P, Zhang T, Qiu C, Sui H (2022). Isoalantolactone enhances the antitumor activity of doxorubicin by inducing reactive oxygen species and DNA damage. Front Oncol.

[49] Ding Y, Wang T, Chen T, Xie C, Zhang Q (2020). Sesquiterpenoids isolated from the flower of Inula japonica as potential antitumor leads for intervention of paclitaxel-resistant non-small-cell lung cancer. Bioorg Chem.

[50] Yu H, Lin L, Zhang Z, Zhang H, Hu H (2020). Targeting NF-κB pathway for the therapy of diseases: mechanism and clinical study. Signal Transduct Target Ther.

[51] Karin M (2006). Nuclear factor-κB in cancer development and progression. Nature.

[52] Di W, Khan M, Rasul A, Sun M, Sui Y, Zhong L (2014). Isoalantolactone inhibits constitutive NF-κB activation and induces reactive oxygen species-mediated apoptosis in osteosarcoma U2OS cells through mitochondrial dysfunction. Oncol Rep.

[53] Liu J, Yang Z, Kong Y, He Y, Xu Y, Cao X (2019). Antitumor activity of alantolactone in lung cancer cell lines NCI-H1299 and Anip973. J Food Biochem.

[54] He Y, Cao X, Kong Y, Wang S, Xia Y, Bi R (2019). Apoptosis-promoting and migration-suppressing effect of alantolactone on gastric cancer cell lines BGC-823 and SGC-7901 via regulating p38MAPK and NF-κB pathways. Hum Exp Toxicol.

[55] Feng Y, Xia J, Xu X, Zhao T, Tan Z, Wang Q (2021). Sesquiterpene lactone Bigelovin induces apoptosis of colon cancer cells through inducing IKK-β degradation and suppressing nuclear factor kappa B activation. Anticancer Drugs.

[56] Roozbehani M, Abdolmohammadi MH, Hamzeloo-Moghadam M, Irani S, Fallahian F (2021). Gaillardin, a potent sesquiterpene lactone induces apoptosis via down-regulation of NF-κβ in gastric cancer cells, AGS and MKN45. J Ethnopharmacol.

[57] Shi K, Liu X, Du G, Cai X, Zhan Y (2020). In vivo antitumour activity of Britanin against gastric cancer through nuclear factor-κB-mediated immune response. J Pharm Pharmacol.

[58] Li K, Zhou Y, Chen Y, Zhou L, Liang J (2020). A novel natural product, britanin, inhibits tumor growth of pancreatic cancer by suppressing nuclear factor-κB activation. Cancer Chemother Pharmacol.

[59] Li X, Yang X, Liu Y, Gong N, Yao W, Chen P (2013). Japonicone A suppresses growth of burkitt lymphoma cells through its effect on NF-κB. Clin Cancer Res.

[60] Chun JK, Seo D-W, Ahn SH, Park JH, You J-S, Lee C-H (2007). Suppression of the NF-kB signalling pathway by ergolide, sesquiterpene lactone. HeLa cells J Pharm Pharmacol.

[61] Song YJ, Lee DY, Kang D-W, Kim YK, Kim S-N, Lee KR (2005). Apoptotic potential of sesquiterpene lactone ergolide through the inhibition of NF-κB signaling pathway. J Pharm Pharmacol.

[62] Ma J-h, Qin L, Li X (2020). Role of STAT3 signaling pathway in breast cancer. Cell Commun Signal.

[63] Chun J, Song K, Kim YS (2018). Sesquiterpene lactones-enriched fraction of Inula helenium L induces apoptosis through inhibition of signal transducers and activators of transcription 3 signaling pathway in MDA-MB-231 breast cancer cells. Phytother Res.

[64] Ahmad B, Gamallat Y, Su P, Husain A, Rehman AU, Zaky MY (2020). Alantolactone induces apoptosis in THP-1 cells through STAT3, survivin inhibition, and intrinsic apoptosis pathway. Chem Biol Drug Des.

[65] Zhang H, Kuang S, Wang Y, Sun X, Gu Y, Hu L (2015). Bigelovin inhibits STAT3 signaling by inactivating JAK2 and induces apoptosis in human cancer cells. Acta Pharmacol Sin.

[66] Fang JY, Richardson BC (2005). The MAPK signalling pathways and colorectal cancer. Lancet Oncol.

[67] Kim MY, Lee H, Ji SY, Kim SY, Hwangbo H, Park S-H (2021). Induction of apoptosis by isoalantolactone in human hepatocellular carcinoma Hep3B cells through activation of the ROS-Dependent JNK signaling pathway. Pharmaceutics.

[68] Pan M-H, Chiou Y-S, Cheng A-C, Bai N, Lo C-Y, Tan D (2007). Involvement of MAPK, Bcl-2 family, cytochrome c, and caspases in induction of apoptosis by 1,6-O, O-diacetylbritannilactone in human leukemia cells. Mol Nutr Food Res.

[69] Glaviano A, Foo ASC, Lam HY, Yap KCH, Jacot W, Jones RH (2023). PI3K/AKT/mTOR signaling transduction pathway and targeted therapies in cancer. Mol Cancer.

[70] Kang X, Wang H, Li Y, Xiao Y, Zhao L, Zhang T (2019). Alantolactone induces apoptosis through ROS-mediated AKT pathway and inhibition of PINK1-mediated mitophagy in human HepG2 cells. Artif Cells Nanomed Biotechnol.

[71] Lee KM, Shin JM, Chun J, Song K, Nho CW, Kim YS (2019). Igalan induces detoxifying enzymes mediated by the Nrf2 pathway in HepG2 cells. J Biochem Mol Toxicol.

[72] Peng Z, Wang Y, Fan J, Lin X, Liu C, Xu Y (2017). Costunolide and dehydrocostuslactone combination treatment inhibit breast cancer by inducing cell cycle arrest and apoptosis through c-Myc/p53 and AKT/14–3–3 pathway. Sci Rep.

[73] Cai H, Li L, Jiang J, Zhao C, Yang C (2019). Costunolide enhances sensitivity of K562/ADR chronic myeloid leukemia cells to doxorubicin through PI3K/Akt pathway. Phytother Res.

[74] Lee J (2009). The sesquiterpene lactone eupatolide sensitizes breast cancer cells to TRAIL through down-regulation of c-FLIP expression. Oncol Rep.

[75] Yang L, Xie J, Almoallim HS, Alharbi SA, Chen Y (2021). Tomentosin inhibits cell proliferation and induces apoptosis in MOLT-4 leukemia cancer cells through the inhibition of mTOR/PI3K/Akt signaling pathway. J Biochem Mol Toxicol.

[76] Arfin S, Jha NK, Jha SK, Kesari KK, Ruokolainen J, Roychoudhury S (2021). Oxidative stress in cancer cell metabolism. Antioxidants.

[77] Gorrini C, Harris IS, Mak TW (2013). Modulation of oxidative stress as an anticancer strategy. Nat Rev Drug Discovery.

[78] Levings DC, Wang X, Kohlhase D, Bell DA, Slattery M (2018). A distinct class of antioxidant response elements is consistently activated in tumors with NRF2 mutations. Redox Biol.

[79] He W, Cao P, Xia Y, Hong L, Zhang T, Shen X (2019). Potent inhibition of gastric cancer cells by a natural compound via inhibiting TrxR1 activity and activating ROS-mediated p38 MAPK pathway. Free Radic Res.

[80] Zhang J, Li Y, Duan D, Yao J, Gao K, Fang J (2016). Inhibition of thioredoxin reductase by alantolactone prompts oxidative stress-mediated apoptosis of HeLa cells. Biochem Pharmacol.

[81] Ding Y, Wang H, Niu J, Luo M, Gou Y, Miao L (2016). Induction of ROS overload by alantolactone prompts oxidative dna damage and apoptosis in colorectal cancer cells. Int J Mol Sci.

[82] Nakatani K, Maehama T, Nishio M, Otani J, Yamaguchi K, Fukumoto M (2021). Alantolactone is a natural product that potently inhibits YAP1/TAZ through promotion of reactive oxygen species accumulation. Cancer Sci.

[83] Xu X, Huang L, Zhang Z, Tong J, Mi J, Wu Y (2019). Targeting non-oncogene ROS pathway by alantolactone in B cell acute lymphoblastic leukemia cells. Life Sci.

[84] Maryam A, Mehmood T, Zhang H, Li Y, Khan M, Ma T (2017). Alantolactone induces apoptosis, promotes STAT3 glutathionylation and enhances chemosensitivity of A549 lung adenocarcinoma cells to doxorubicin via oxidative stress. Sci Rep.

[85] Jang DK, Lee I-S, Shin H-S, Yoo HM (2020). 2α-hydroxyeudesma-4,11(13)-dien-8β,12-olide isolated from *Inula britannica* induces apoptosis in diffuse large B-cell lymphoma cells. Biomolecules.

[86] Seo JY, Lim SS, Kim JR, Lim J-S, Ha YR, Lee IA (2008). Nrf2-mediated induction of detoxifying enzymes by alantolactone present inInula helenium. Phytother Res.

[87] Chen J, Zhang Y, Huang R, Cao L, Zhang Y, Lian M (2022). Alantolactone inhibits oesophageal adenocarcinoma cells through nuclear factor erythroid 2-related factor 2-mediated reactive oxygen species increment. Basic Clin Pharmacol Toxicol.

[88] Hu Z, Qin J, Zhang H, Wang D, Hua Y, Ding J (2012). Japonicone A antagonizes the activity of TNF-α by directly targeting this cytokine and selectively disrupting its interaction with TNF receptor-1. Biochem Pharmacol.

[89] Bailly C, Vergoten G (2022). Japonicone A and related dimeric sesquiterpene lactones: molecular targets and mechanisms of anticancer activity. Inflamm Res.

[90] Deng L, Meng T, Chen L, Wei W, Wang P (2020). The role of ubiquitination in tumorigenesis and targeted drug discovery. Signal Transduct Target Ther.

[91] Qi S, Guan X, Zhang J, Yu D, Yu X, Li Q (2022). Targeting E2 ubiquitin-conjugating enzyme UbcH5c by small molecule inhibitor suppresses pancreatic cancer growth and metastasis. Mol Cancer.

[92] Liu L, Hua Y, Wang D, Shan L, Zhang Y, Zhu J (2014). A sesquiterpene lactone from a medicinal herb inhibits proinflammatory activity of TNF-α by inhibiting ubiquitin-conjugating enzyme UbcH5. Chem Biol.

[93] Chen H, Wu G, Gao S, Guo R, Zhao Z, Yuan H (2017). Discovery of potent small-molecule inhibitors of ubiquitin-conjugating enzyme UbcH5c from α-santonin derivatives. J Med Chem.

[94] Wade M, Li Y-C, Wahl GM (2013). MDM2, MDMX and p53 in oncogenesis and cancer therapy. Nat Rev Cancer.

[95] Qin J-J, Wang W, Voruganti S, Wang H, Zhang W-D, Zhang R (2015). Identification of a new class of natural product MDM2 inhibitor: in vitro and in vivo anti-breast cancer activities and target validation. Oncotarget.

[96] Wu G, Zhu L, Yuan X, Chen H, Xiong R, Zhang S (2017). Britanin ameliorates cerebral ischemia-reperfusion injury by inducing the Nrf2 protective pathway. Antioxid Redox Signal.

[97] Sánchez-Ortega M, Carrera AC, Garrido A (2021). Role of NRF2 in lung cancer. Cells.

[98] Menegon S, Columbano A, Giordano S (2016). The dual roles of NRF2 in cancer. Trends Mol Med.

[99] Sharma BR, Kanneganti T-D (2021). NLRP3 inflammasome in cancer and metabolic diseases. Nat Immunol.

[100] Ren M, Chen J, Xu H, Li W, Wang T, Chi Z (2023). Ergolide covalently binds NLRP3 and inhibits NLRP3 inflammasome-mediated pyroptosis. Int Immunopharmacol.

[101] Moossavi M, Parsamanesh N, Bahrami A, Atkin SL, Sahebkar A (2018). Role of the NLRP3 inflammasome in cancer. Mol Cancer.

[102] Song B, Liu XS, Rice SJ, Kuang S, Elzey BD, Konieczny SF (2013). Plk1 phosphorylation of Orc2 and Hbo1 contributes to gemcitabine resistance in pancreatic cancer. Mol Cancer Ther.

[103] Alam P, Tyagi R, Farah MA, Rehman MT, Hussain A, AlAjmi MF (2021). Cytotoxicity and molecular docking analysis of racemolactone I, a new sesquiterpene lactone isolated from *Inula racemosa*. Pharm Biol.

[104] Agashe RP, Lippman SM, Kurzrock R (2022). JAK: Not just another kinase. Mol Cancer Ther.

[105] O’Donnell JS, Teng MWL, Smyth MJ (2018). Cancer immunoediting and resistance to T cell-based immunotherapy. Nat Rev Clin Oncol.

[106] Tang Q, Chen Y, Li X, Long S, Shi Y, Yu Y (2022). The role of PD-1/PD-L1 and application of immune-checkpoint inhibitors in human cancers. Front Immunol.

[107] Wu Q, Jiang L, Li S, He Q, Yang B, Cao J (2020). Small molecule inhibitors targeting the PD-1/PD-L1 signaling pathway. Acta Pharmacol Sin.

[108] Zhang YF, Zhang ZH, Li MY, Wang JY, Xing Y, Ri M (2021). Britannin stabilizes T cell activity and inhibits proliferation and angiogenesis by targeting PD-L1 via abrogation of the crosstalk between Myc and HIF-1α in cancer. Phytomedicine.

[109] Mahalingam D, Peguero J, Cen P, Arora SP, Sarantopoulos J, Rowe J (2019). A phase II, multicenter, single-arm study of mipsagargin (G-202) as a second-line therapy following sorafenib for adult patients with progressive advanced hepatocellular carcinoma. Cancers.

[110] Hu X, Qi C, Feng F, Wang Y, Di T, Meng Y (2022). Combining network pharmacology, RNA-seq, and metabolomics strategies to reveal the mechanism of Cimicifugae Rhizoma—Smilax glabra Roxb herb pair for the treatment of psoriasis. Phytomedicine.

[111] Fedorov II, Lineva VI, Tarasova IA, Gorshkov MV (2022). Mass spectrometry-based chemical proteomics for drug target discoveries. Biochemistry.

[112] Tu Y, Tan L, Tao H, Li Y, Liu H (2023). CETSA and thermal proteome profiling strategies for target identification and drug discovery of natural products. Phytomedicine.

[113] Chavanieu A, Pugnière M (2016). Developments in spr fragment screening. Expert Opin Investig Drugs.

[114] Han Y-Y, Tang J-J, Gao R-F, Guo X, Lei M, Gao J-M (2016). A new semisynthetic 1- O -acetyl-6- O -lauroylbritannilactone induces apoptosis of human laryngocarcinoma cells through p53-dependent pathway. Toxicol In Vitro.

[115] Liu B, Han M, Sun R-H, Wang J-J, Zhang Y-P, Zhang D-Q (2010). ABL-N-induced apoptosis in human breast cancer cells is partially mediated by c-Jun NH2-terminal kinase activation. Breast Cancer Res.

[116] Chun J, Park S-M, Lee M, Ha IJ, Jeong M-K (2023). The sesquiterpene lactone-rich fraction of *Inula helenium* L. enhances the antitumor effect of anti-PD-1 antibody in colorectal cancer: integrative phytochemical, transcriptomic, and experimental analyses. Cancers.

[117] Karami A, Hamzeloo-Moghadam M, Yami A, Barzegar M, Mashati P, Gharehbaghian A (2019). Antiproliferative effect of Gaillardin from *Inula oculus-christi* in human leukemic cells. Nutr Cancer.

[118] Li M, Song L-H, Yue GG-L, Lee JK-M, Zhao L-M, Li L (2017). Bigelovin triggered apoptosis in colorectal cancer in vitro and in vivo via upregulating death receptor 5 and reactive oxidative species. Sci Rep.

[119] Wang B, Nie C-H, Xu J, Wan D-L, Xu X, He J-J (2023). Bigelovin inhibits hepatocellular carcinoma cell growth and metastasis by regulating the MAPT-mediated Fas/FasL pathway. Neoplasma.

[120] Bocca C, Gabriel L, Bozzo F, Miglietta A (2004). A sesquiterpene lactone, costunolide, interacts with microtubule protein and inhibits the growth of MCF-7 cells. Chem Biol Interact.

[121] Boldbaatar A, Lee S, Han S, Jeong A, Ka H, Buyanravjikh S (2017). Eupatolide inhibits the TGF-β1-induced migration of breast cancer cells via downregulation of SMAD3 phosphorylation and transcriptional repression of ALK5. Oncol Lett.

[122] Ma X, Wu K, Xu A, Jiao P, Li H, Xing L (2021). The sesquiterpene lactone eupatolide induces apoptosis in non-small cell lung cancer cells by suppressing STAT3 signaling. Environ Toxicol Pharmacol.

[123] Lee C, Lee J, Nam M, Choi Y, Park S-H (2019). Tomentosin displays anti-carcinogenic effect in human osteosarcoma MG-63 cells via the induction of intracellular reactive oxygen species. Int J Mol Sci.

[124] Yang H, Zhao H, Dong X, Yang Z, Chang W (2020). Tomentosin induces apoptotic pathway by blocking inflammatory mediators via modulation of cell proteins in AGS gastric cancer cell line. J Biochem Mol Toxicol.

